# Microstate Analysis of Continuous Infant EEG: Tutorial and Reliability

**DOI:** 10.1007/s10548-024-01043-5

**Published:** 2024-03-02

**Authors:** Armen Bagdasarov, Denis Brunet, Christoph M. Michel, Michael S. Gaffrey

**Affiliations:** 1 Department of Psychology & Neuroscience, Duke University, Reuben-Cooke Building, 417 Chapel Drive, Durham, NC 27708 USA; 2 Department of Basic Neurosciences, University of Geneva, Campus Biotech, 9 Chemin des Mines, Geneva, 1202 Switzerland; 3 Center for Biomedical Imaging (CIBM) Lausanne, EPFL AVP CP CIBM Station 6, Lausanne, 1015 Switzerland; 4 Children’s Wisconsin, 9000 W. Wisconsin Avenue, Milwaukee, WI 53226 USA; 5 Medical College of Wisconsin, Division of Pediatric Psychology and Developmental Medicine, Department of Pediatrics, 8701 Watertown Plank Road, Milwaukee, WI 53226 USA

**Keywords:** Microstates, Resting-state, Source localization, Infants, Tutorial, Reliability

## Abstract

Microstate analysis of resting-state EEG is a unique data-driven method for identifying patterns of scalp potential topographies, or microstates, that reflect stable but transient periods of synchronized neural activity evolving dynamically over time. During infancy – a critical period of rapid brain development and plasticity – microstate analysis offers a unique opportunity for characterizing the spatial and temporal dynamics of brain activity. However, whether measurements derived from this approach (e.g., temporal properties, transition probabilities, neural sources) show strong psychometric properties (i.e., reliability) during infancy is unknown and key information for advancing our understanding of how microstates are shaped by early life experiences and whether they relate to individual differences in infant abilities. A lack of methodological resources for performing microstate analysis of infant EEG has further hindered adoption of this cutting-edge approach by infant researchers. As a result, in the current study, we systematically addressed these knowledge gaps and report that most microstate-based measurements of brain organization and functioning except for transition probabilities were stable with four minutes of video-watching resting-state data and highly internally consistent with just one minute. In addition to these results, we provide a step-by-step tutorial, accompanying website, and open-access data for performing microstate analysis using a free, user-friendly software called Cartool. Taken together, the current study supports the reliability and feasibility of using EEG microstate analysis to study infant brain development and increases the accessibility of this approach for the field of developmental neuroscience.

**Supplementary Information:**

The online version contains supplementary material available at 10.1007/s10548-024-01043-5.

## Introduction

Infancy is a key period of human development during which the neurobiological foundations of emerging social, emotional, and cognitive skills are shaped through the interaction of child-specific factors and environmental experiences (Gabard-Durnam and McLaughlin 2020; Johnson 2001; Nelson et al. 2007; Paterson et al. 2006). Therefore, reliably characterizing the functional properties of brain activity and organization during infancy provides a unique opportunity for understanding the impact of early life experiences on brain development and associated behavior (Lopez et al. 2023). Toward this end, electroencephalography (EEG) has been routinely used as a direct, non-invasive, and low-cost measure of brain activity that can be collected from infants during various states of arousal and/or activity (Azhari et al. 2020; Bell and Cuevas 2012). Most commonly, given its ease of acquisition and tolerance of head and body movement, EEG is readily and frequently acquired from infants while they sit on their caregiver’s lap and watch relaxing videos. Prior research using EEG data from awake infants during video-watching have tended to focus on single metrics of global (e.g., total power or power spectral density analyses) or local (e.g., region-of-interest power or event-related potential analyses) features (Braithwaite et al. 2020; Jones et al. 2020). However, recent data indicates that understanding the associations between functional brain networks (i.e., whole-brain dynamics; Xie et al. 2022) and behavior are crucial for advancing understanding of brain development during the first years of life. One promising but relatively unexplored method for characterizing whole-brain dynamics collected from high-density EEG during infancy is microstate analysis.

Microstate analysis is a data-driven approach for identifying patterns of scalp potential topographies, or *microstates*, that reflect very short periods (i.e., typically less than ~ 150 ms) of synchronized neural activity (i.e., large-scale functional networks) evolving dynamically over time (Khanna et al. 2015; Michel and Koenig 2018). A small number of four to seven canonical topographies have been replicated and consistently shown to explain the majority of topographic variance in the entire EEG signal recorded during rest (i.e., the absence of external task demands) in both children and adults. Several temporal properties are also frequently calculated for each microstate and have been reported to show unique variation in their values and associations with individual differences in behavior. Temporal measures routinely used in studies include (1) global explained variance (GEV), the total variance in the data explained by a microstate, (2) duration, the average time in milliseconds (ms) that a microstate was present before transitioning to another microstate, (3) coverage, the percentage of time for which a microstate was present, (4) occurrence, the frequency with which a microstate was present per second, and (5) transition probabilities, the probability of one microstate coming after another in the sequence. Importantly, the neural generators for each microstate can be identified with source localization techniques, a critical step in understanding their functional significance and potential relevance to developing behavior.

While microstate analysis has proven to be a highly informative method for studying brain function and organization at the millisecond-level in adults, very few studies using this approach in infants have been published. More specifically, of the seven publications that we identified, four used the microstate analytic approach to examine event-related data (Bucsea et al. 2023; Gui et al. 2021; Maitre et al. 2020; Rupawala et al. 2023), two examined microstates during sleep (Hermans et al. 2023; Khazaei et al. 2021), and one used microstate analysis to examine spontaneous EEG data collected from infants (i.e., 34, 6-10-month-olds) during video-watching (Brown and Gartstein 2023). Unfortunately, none of this prior work investigated the reliability of microstate-related measures at this age, a critical step in understanding the potential use of microstates to study individual differences in behavior and development (Lopez et al. 2023). However, previous work has demonstrated the reliability of microstate analysis in adults and suggests it is likely present in younger age groups as well. More specifically, in adults, prior studies have indicated good-to-excellent internal consistency (i.e., stability of temporal properties within the same session) and short- and long-term test-retest reliability (i.e., stability of temporal properties between multiple sessions recorded in the same week) for each temporal property of each identified microstate (Antonova et al. 2022; Khanna et al. 2014; Kleinert et al. 2023; Liu et al. 2020; Popov et al. 2023). Notably, Liu et al. (2020) demonstrated that as little as 1–2 min of data showed sufficient psychometric properties for GEV, duration, coverage, and occurrence values (i.e., intraclass correlations (ICCs) > 0.60). Recently, Kleinert et al. (2023) demonstrated good-to-excellent short-term (ICCs = 0.87-0.92) and long-term (ICCs = 0.67-0.85) test-retest reliability of duration, coverage, and occurrence. Transition probabilities, however, have been shown to be much less reliable than GEV, duration, coverage, and occurrence values (Antonova et al. 2022; Kleinert et al. 2023; Liu et al. 2020). Critically, strong reliability has been demonstrated across microstate clustering algorithms (Khanna et al. 2014), recording lengths (two vs. three minutes; Kleinert et al. 2023), and EEG channel densities (Khanna et al. 2014; Kleinert et al. 2023; Zhang et al. 2021); though Zhang et al. (2021) demonstrated 8- and 19-channel arrays to have significantly lower reliability than higher density arrays. While previous research has indicated high reliability of resting-state EEG source localization with approximately 1.5-2 minutes of data (Cannon et al. 2012), no research exists examining the reliability of microstate sources at any age. Taken together, while studies in adults indicate strong promise for the reliability of microstate analysis in EEG data collected from infants, equal reliability cannot be assumed across developmental stages as shown in Popov et al. (2023), and must be individually examined for each population. Indeed, previous work using other EEG approaches such as functional connectivity (e.g., phase lag index) and event-related potentials has demonstrated that different quantities of data may be needed for reliable estimates of brain-based measures during infancy (Haartsen et al. 2020; Munsters et al. 2019).

One barrier that may be contributing to the dearth of published studies using the microstate analytic approach for characterizing infant EEG data is the lack of comprehensive, step-by-step methodological resources for infant researchers to employ this approach in their own work. Resources that specifically use examples from infant EEG data are more likely to be adopted by infant EEG researchers than resources that focus on other populations. Production of resources for analyzing EEG data in ways that inform understanding of brain function and organization (such as microstate analysis) are especially important as large-scale, multi-site, longitudinal infant EEG studies such as the HEALthy Brain and Child Development Study (Jordan et al. 2020), Bucharest Early Intervention Project (Zeanah et al. 2003), Bangladesh Early Adversity Neuroimaging Study (Turesky et al. 2019), Safe Passage Study (Dukes et al. 2014), YOUth Cohort Study (Onland-Moret et al. 2020), Eurosibs Consortium (Jones et al. 2019), and Baby Siblings Research Consortium (Levin et al. 2017) are amidst or have completed data collection, with opportunities for data access and analysis. And, in line with the open science movement, sharing of data and analytic methods will be critical for the replication of findings. Another potential explanation for the paucity of microstate studies during infancy is the lack of adaptation of the microstate analytic method for use with EEG data from infants. For example, current and popular tools for performing microstate analysis (e.g., Cartool) do not include age-appropriate MRI brain templates for the source localization of microstates. Whether the microstate analytic method requires infant-specific changes for how microstates and their temporal properties are identified and measured also remains unknown.

As a first step toward validating the use of EEG microstates for investigating infant brain development, the current study explored the feasibility of identifying microstates during video-watching resting-state and examined their psychometric reliability in 48, 5-10-month-old infants using high-density EEG. Specifically, we assessed (1) the stability of microstate topographies, their temporal properties, their transition probabilities, and their neural sources with increasing EEG data durations (i.e., 1–5 min), and (2) the internal consistency (i.e., split-half reliability) of the temporal properties, transition probabilities, and neural sources at each data duration. Given the lack of studies examining resting-state microstates during infancy, we did not make specific predictions about microstate characteristics (i.e., topographies, temporal properties, transition probabilities, neural sources) or their reliability. In order to facilitate methodological access to microstate analysis, the current study also provides resources for analyzing microstates during infancy in line with recent efforts to maximize the potential of EEG as a developmental neuroscience tool (Buzzell et al. 2023). Toward this end, we have provided a step-by-step tutorial, accompanying website, and required files (e.g., age-appropriate MRI brain templates) for performing microstate analysis and microstate source localization of EEG data using Cartool software (Brunet et al. 2011). We also shared our EEG data in the Brain Imaging Data Structure (BIDS; Pernet et al. 2019) format on OpenNeuro (Markiewicz et al. 2021).

## Methods

### Participants

Participants were 48, 5-10-month-old infants (27 or 56.25% male). Recruitment and demographic details are described in Supplementary Materials S1 and S2, respectively. All research was approved by the Duke University Health System Institutional Review Board and carried out in accordance with the Declaration of Helsinki. Caregivers provided informed consent, and compensation was provided for their participation.

### EEG data Acquisition, Preprocessing, and Quality Checks

Infants sat on their caregiver’s lap and watched up to 15 minutes of dynamic, relaxing videos with sound (i.e., 10, 90-second videos separated by breaks during which caregivers could play with their infant; information about the videos are provided in Supplementary Materials S3). Before each video started, an attention grabber (i.e., three-second video of a noisy rattle) directed the infant’s attention to the screen. Videos were presented with E-Prime software (Psychological Software Tools, Pittsburgh, PA). Caregivers were instructed to silently sit still during videos. If infants shifted their attention away from the screen, caregivers were permitted to re-direct their attention only by pointing to the screen. EEG was recorded at 1000 Hz (Hz) and referenced to the vertex (channel Cz) using a 128-channel HydroCel Geodesic Sensor Net (Electrical Geodesics, Eugene, OR). Impedances were maintained below 50 kilohms throughout the EEG session.

Offline preprocessing was performed with EEGLAB (Delorme and Makeig 2004) in MATLAB (MathWorks, Natick, MA). Details with instructions for their use are available on https://github.com/gaffreylab/EEG-Microstate-Analysis-Tutorial. Briefly, 24 outer ring channels that often contain a large amount of artifact in infant data were removed due to their location near the base of the skull or on the neck or face. Data were downsampled to 250 Hz, and bandpass filtered 1–20 Hz. A 20 Hz cutoff was chosen to minimize the impact of high frequency noise on the data, and this cutoff has been used in other published microstate studies (Koenig et al. 2002; Milz et al. 2016). Periods of inattention were manually rejected based on session notes. Bad channels were removed and interpolated using spherical splines if they were (1) flat for more than five seconds, (2) contained more than four standard deviations of line noise relative to all other channels, or (3) correlated at less than 0.80 to surrounding channels. Following, data were re-referenced to the average. Then, a copy of the data was created, cleaned using Artifact Subspace Reconstruction (ASR; Mullen et al. 2015; i.e., artifacted periods were removed using a burst criterion of 20 as recommended by Chang et al. 2020), and submitted to extended infomax independent component analysis (ICA; Lee et al. 1999) with principal component analysis dimensionality reduction (i.e., 50 components). The ICLabel plugin was used to flag components that had a probability of at least 0.70 of being related to eye activity, muscle activity, heart artifacts, line noise, or channel noise (Pion-Tonachini et al. 2019). The resulting ICA fields and flags were subsequently copied over to the full-length data before ASR was performed, and flagged ICA components were removed. Application of the ICA matrix to the full-length data and subsequent removal of independent components allowed for the preservation of data that would have otherwise been removed by ASR or other artifact removal methods. Data was segmented into nonoverlapping one-second epochs and were removed in two steps using the TBT plugin (Ben-Shachar 2018). First, to remove data containing residual eye-related artifacts, epochs were removed if any one of six frontal channels contained amplitudes > 150 or < -150 µV as recommended for infant data by Debnath et al. (2020). Next, epochs were removed if at least 10 channels contained (1) amplitudes > 150 or < -150 µV (Debnath et al. 2020), (2) joint probabilities (i.e., probabilities of plausible activity) above 3 standard deviations for local or global thresholds as performed in our previous work (Bagdasarov et al. 2022) and suggested by the work of others (Gabard-Durnam et al. 2018), or (3) a > 100 µV maximum-minimum amplitude difference between data samples. To avoid rejecting epochs that contained only a small number of noisy channels, if less than 10 channels met rejection criteria, representing < 10% of the total number of channels, the epoch was not removed, but the channels were interpolated for that epoch only. Lastly, data was re-referenced to the average a final time.

Data quality was rigorously assessed. Participants who did not pass EEG data quality checks were excluded from further analyses (see Supplementary Materials S4). Also, participants with less than five minutes of EEG data passing initial preprocessing and subsequent quality control were excluded so that reliability analyses could be conducted (see below). After removal of participants, data from the final pool of participants was cut to one-, two-, three-, four-, and five-minute durations, resulting in five data files per participant.

### Microstate Analysis

#### Tutorial and Data Availability

Microstate analysis was performed in Cartool, a free, easy-to-use, publicly available, and soon-to-be open-source software that does not have any software dependencies (Brunet et al. 2011). Cartool is programmed by Denis Brunet, from the Functional Brain Mapping Laboratory (FBMLab), Geneva, Switzerland, and is supported by the Center for Biomedical Imaging (CIBM) of Geneva and Lausanne. Cartool includes documentation for all processes performed here. We add to this documentation by providing a step-by-step tutorial and accompanying website for performing microstate analysis: https://github.com/gaffreylab/EEG-Microstate-Analysis-Tutorial. Importantly, while the current paper is focused on microstate analysis of data collected from infants, this tutorial is applicable to data collected from most human populations. Our tutorial includes (1) instructions for data preprocessing, including MATLAB scripts, (2) instructions for performing all stages of microstate analysis, including information about data preparation (e.g., formatting the EEG data, creating a compatible channel locations file) and source localization (e.g., template MRI files), (3) raw and preprocessed data files that are openly shared in BIDS format on OpenNeuro (OpenNeuro Accession Number: ds004635; https://openneuro.org/datasets/ds004635), and (4) outputs of all statistical analyses performed to assess the reliability of microstates as described below. Moreover, step-by-step instructions are accompanied by screenshots and video recordings to increase accessibility.

In this tutorial, microstate analysis was performed in three stages: individual-level clustering, group-level clustering, and backfitting. Then, source localization was performed to identify the neural generators of each microstate. Before beginning the first stage, however, a spatial filter was applied to the data (Michel and Brunet 2019). This filter, which is unique to Cartool and particularly beneficial for source localization, helped increase the signal-to-noise of the data by removing topographic outliers and smoothing topographies at each time point of the preprocessed data. Further, since reliability analyses were performed for each of five data durations (i.e., one, two, three, four, and five minutes), microstate and source analyses were performed five times.

#### Stage 1: Individual-Level Clustering

At the individual-level (i.e., for each participant’s data), topographies at global field power (GFP) peaks representing timepoints of the highest signal-to-noise ratio were extracted (Brunet et al. 2011). Fifty epochs, each composed of a random subsample of the extracted topographies and representing 99.9% of the participant’s data, were submitted to a polarity-invariant modified *k*-means cluster analysis (Pascual-Marqui et al. 1995), which was set to repeat 50 times and identify 1–12 clusters of topographies for each epoch (see Supplementary Materials S5 for number of subsamples, which varied, for each data duration). The resampling approach is thought to improve the reliability of *k*-means clustering and has been used in recent work (Bagdasarov et al. 2022, 2023; Férat et al. 2022). The meta-criterion – an aggregate measure of six independent criteria (Bréchet et al. 2019; Custo et al. 2017) – determined the optimal number of clusters for each epoch, resulting in *k* optimal clusters for each of 50 epochs or k*50 topographies for each participant.

#### Stage 2: Group-Level Clustering

At the group-level (i.e., for data from the group of participants), the 50 epochs of *k* optimal clusters from each participant were combined, resulting in 50*k topographies for each of 48 participants or 50*k*48 topographies for the group. One-hundred epochs, each composed of a random subsample of these topographies and representing 99.9% of the group’s 50*k*48 topographies were submitted to a polarity-invariant modified *k*-means cluster analysis, which was set to repeat 100 times and identify 1–15 clusters of topographies for each epoch. The meta-criterion determined the optimal number of clusters for each epoch, resulting in *k* optimal clusters for each of 100 epochs or k*100 topographies. These topographies were combined and submitted to a final *k*-means cluster analysis with the same parameters, and the meta-criterion was used as guidance from which we selected the optimal number of clusters based on resting-state topographies observed in prior work (see below); now, the group-level microstates.

For analysis of resting-state data, Cartool’s built-in Reference Guide recommends use of the meta-criterion automatically at the individual-level; that is, the meta-criterion should select the optimal number of clusters for each participant. However, at the group-level, it is recommended to use the meta-criterion as guidance from which the researcher must interpret its suggestions based on previous research, their experience, and the type of data they are working with. For example, if the meta-criterion clearly shows a solution with a single maximum position, then the researcher should use that solution as the optimal one. However, if the meta-criterion shows multiple local maxima, the researcher must decide which solution is optimal rather than simply selecting the one with the highest value. The tutorial GitHub page provides a step-by-step guide for selecting the optimal number of microstates using the meta-criterion.

#### Stage 3: Backfitting

The last stage of microstate analysis – backfitting – was performed on each participant’s preprocessed, spatially-filtered data, and resulted in rich temporal information characterizing each group-level microstate for each participant. First, each participant’s data was normalized by the median of GFP to account for individual differences in scalp potential due to varying skull conductivity. Then, each time point of each participant’s data was labeled with one group-level microstate; the one that was most spatially correlated with the topography at that time point (i.e., winner-take-all approach). The polarity of topographies was ignored when calculating the correlation and the minimum correlation for time points assigned to a microstate was 0.50. After backfitting, temporal smoothing was applied (window half-size of 32 ms and Besag factor of 10; Pascual-Marqui et al. 1995), and improbably small segments were removed, such that segments smaller than 32 ms were divided in half with the first half added to the preceding segment and the second half added to the proceeding segment. Lastly, the GEV, duration, coverage, and occurrence values of each microstate were calculated for each participant, as well as first-order Markov chain transition probabilities (expected and observed). Transition probabilities for each transition direction were normalized to account for the occurrence of microstates, which varied by participant, by dividing observed probability values by expected probability values.

#### Stage 4: Source Localization

After backfitting, microstates were organized by cluster into separate files for each participant, and source localization was performed for each cluster separately. Two sets of T1-weighted template MRI files (5-8-month-old and 8-11-month-old templates) were obtained from the National Institutes of Health-funded MRI Study of Normal Brain Development (Fonov et al. 2009). Downloaded files included the head (brain with skull), the brain (skull-stripped), and grey matter (extracted from brain). In addition, for each set of files, tissues were segmented in Cartool to identify the following tissue boundaries: scalp, fat, muscle, cerebrospinal fluid, blood, eyes, air, skull and three skull tissue types (compact, spongy, suture), brain, and grey and white matter.

Source localization was performed separately for 5-7-month-old (using the 5-8-month-old template files) and 8-10-month-old infants (using the 8-11-month-old template files). For each age group, solution points were distributed equally in a grey matter-constrained head model of the age-appropriate infant MRI brain volume template with a 5 mm voxel resolution (6591 solution points for 5-8-month-old template grey matter and 6768 solution points for 8-11-month-old template grey matter). The 105-channel EEG net template was co-registered to the MRI head model. Sources were modeled using a 4-shell, adaptive local spherical model with anatomical constraints (LSMAC), which built local spheres with different radii for each channel by estimating the thickness of the scalp, skull, cerebrospinal fluid, and brain under each channel, allowing the real geometry between solution points and channels to be accounted for (Brunet et al. 2011). Then, the Low Resolution Brain Electromagnetic Tomography (LORETA; Pascual-Marqui et al. 1994) distributed linear inverse solution was calculated to estimate the strength of activity at each solution point. The results were optimized with regularization, which accounted for background EEG noise and enforced smoothness, and standardized to correct for the variability of EEG power across time, a procedure automatically implemented in Cartool to eliminate activation biases (Michel and Brunet 2019). The amplitude of activity produced at each solution point was saved as a scalar, positive value and averaged across time points for each microstate. Inverse solutions were combined across participants and thresholded to the solution points above the 95th percentile of values as done in previous work (Bagdasarov et al. 2022; Bréchet et al. 2020, 2021). The final product was a thresholded, group-level source distribution for each microstate.

Results of source localization can be viewed in Cartool or other software. Here, sources were viewed in the Analysis of Functional NeuroImages (AFNI; Cox 1996) program, which facilitated the assessment of their reliability (see below). Sources were first converted from two-dimensional to three-dimensional in Cartool by computing intermediate voxels from the grey matter-constrained head model using cubic kernel convolution (no new maxima were artificially created). Then, in AFNI, sources were converted to binary values (0 and 1) using the *3dcalc* command to indicate presence of solution points above the 95th percentile of values or lack thereof at each voxel.

### Reliability Analyses

#### Stability Across Data Durations

We assessed whether microstate topographies, their temporal properties (GEV, duration, coverage, and occurrence values), their transition probabilities, and their neural sources were stable or changed with increasing data durations (one, two, three, four, and five minutes). For each of the five data durations, the number of microstates selected by the meta-criterion and their topographies were compared (topographies were compared visually and with spatial correlations). Whether topographic stability or lack thereof was a function of sample size was also assessed by randomly assigning participants into five nested groups of increasing sample sizes (*n* = 10, 20, 30, 40, and 48). For each group and data duration, additional microstate analyses (only stages 1 and 2) were performed, resulting in 25 additional microstate analyses from which topographies were compared.

Next, to assess the stability of each microstate’s four temporal properties across data durations, each microstate-temporal property combination was submitted to a linear mixed-effects model; data duration (fixed effect) served as the predictor. We included a crossed random intercept for participant and an autoregressive covariance structure to account for dependencies in data duration (i.e., the two-minute chunk of data contained the one-minute chunk, the three-minute chunk contained the two-minute chunk, and so on). We fit the model using the *nlme* package (Pinheiro et al. 2017) in R (R Core Team 2022). The estimation method was maximum likelihood. A Type III Analysis of Variance (ANOVA) with the Kenward-Roger method was performed to assess the significance of the effect of data duration on the dependent variable. Given the large number of models, *p* values for the fixed effect of data duration for each ANOVA were Bonferroni-corrected (Bland and Altman 1995) for the total number of models (i.e., the number of microstates multiplied by the number of temporal properties assessed). To estimate the magnitude of the effect of data duration, partial eta squared (η2) was calculated using the *effectsize* package (Ben-Shachar et al. 2020). The stability of transition probabilities across data durations was similarly assessed for each transition direction using linear mixed-effects models with data duration as a fixed effect and participants as a random effect. Given the large number of models, *p* values for the fixed effect of data duration for each ANOVA were Bonferroni-corrected for the total number of models (i.e., the number of possible transition directions). For ANOVA models with a significant fixed effect of data duration, pairwise differences were estimated using the *emmeans* package (Lenth et al. 2019), and p values were Scheffé-corrected (Ruxton and Beauchamp 2008; Scheffé 1953) for the number of comparisons, which was always ten. For significant pairwise differences, bias-corrected and accelerated 95% confidence intervals of the paired mean differences were calculated by performing nonparametric bootstrap resampling (5000 resamples) using the *dabestr* package (Ho et al. 2019). Further, analyses were performed with and without outliers, which were identified using the *rstatix* package (Kassambara 2020). Boxplots within each data duration determined outliers as values above Quartile 3 + 3*IQR or below Quartile 1–3*IQR (IQR = Interquartile Range).

Lastly, to assess the stability of group-level source localization results across data durations, the Dice similarity coefficient (DSC) was calculated between microstate source distributions from the five data durations in AFNI using the *3dSliceNDice* command. The DSC represents the spatial overlap between source distributions and was calculated on a slice-by-slice basis along each axis of the brain for slices where either or both of the source distributions had at least one nonzero voxel. Then, DSC values were averaged across axes. Since source localization was performed separately for 5-7-month-old and 8-10-month-old infants, the DSC procedure was performed separately as well, and then values were averaged.

#### Internal Consistency

The internal consistency of microstate temporal properties (GEV, duration, coverage, and occurrence values) and transition probabilities (for each transition direction) at each data duration (one, two, three, four, and five minutes) was assessed with Spearman-Brown split-half reliability coefficients in R using the *splithalfr* package (Pronk et al. 2022). During the third stage of microstate analysis, backfitting for each data duration was performed on even and odd segments. Specifically, data for each duration was split into six equal segments (see Supplementary Materials S6 for number of time frames representing each segment for each data duration), and backfitting was performed twice; once on even segments and once on odd segments, resulting in two sets of temporal properties and transition probabilities. The Spearman-Brown split-half reliability coefficient was then calculated between the two sets for all temporal properties and transition probabilities for each microstate and data duration. Analyses were performed with and without multivariate outliers, which were identified using the Minimum Covariance Determinant (MCD; Rousseeuw and Driessen 1999) using the *MASS* package (Ripley et al. 2013). The internal consistency of source localization results at each data duration was assessed with the DCS. Source localization was performed separately on even and odd segments for each data duration from the backfitting procedure described above. Then, the DCS was calculated between the two sets of source distributions for each microstate and data duration. As done above, the DSC procedure was performed separately for source localization results from 5-7-month-old and 8-10-month-old infants, and then values were averaged.

To facilitate the interpretation of internal consistency and DSC stability estimates, values were reported using qualitative descriptors based on previous work using similar thresholds (Lopez et al. 2023): poor (values < 0.40), fair (0.40 ≤ values ≤ 0.59), good (0.60 ≤ values ≤ 0.74), and excellent (values ≥ 0.75).

## Results

### Stability Across Data Durations

**Fig. 1:**
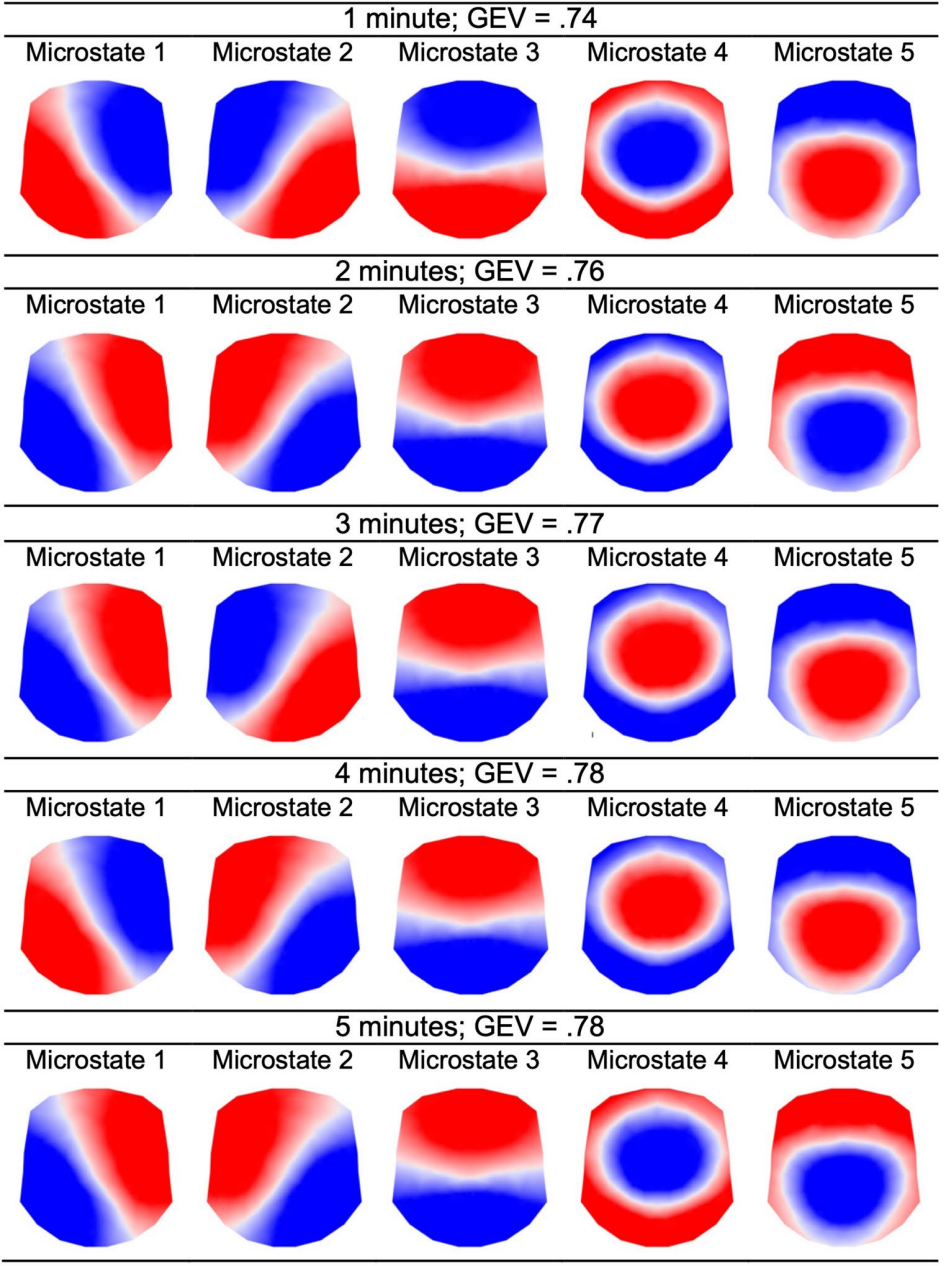
Five infant microstates show similar topographies across varying EEG data durations. *Note*. Microstates are polarity-invariant (i.e., colors do not indicate polarity).

The meta-criterion for determining the optimal number of microstates revealed five microstates labeled 1–5, which explained between 0.74 and 0.78 of GEV at the group-level, for all five data durations (Fig. 1). Microstate topographies were visually very similar (Fig. 1) and highly spatially correlated (correlations all > 0.99) across data durations. Descriptive statistics of microstate temporal properties and transition probabilities are provided in Supplementary Materials S7. Backfitting details are provided in Supplementary Materials S8. Comparing microstate topographies across increasingly larger groups of participants and data durations up to five minutes, results revealed that all data durations and groups of participants showed a five-microstate solution with the exception of the following combinations: (1) one minute of data with 30 participants showed a six-microstate solution, (2) two, four, and five minutes of data with 10 participants showed a six-microstate solution, and (3) three minutes of data with 10 participants showed a five- or six-microstate solution (see Supplementary Materials S9 and tutorial GitHub page for all topographies). Of note, solutions that contained a *transition* state (i.e., topographically appeared to represent the transition between two canonical microstates and was spatially poorly correlated with a canonical microstate) were excluded from being candidates of the optimal solution (see Supplementary Materials S10). That is, while transition states may be meaningful, their interpretation in the context of the current literature is unclear.

Results of linear mixed-effects models are presented for models with outliers removed while models without outliers removed are available on the tutorial GitHub page. No differences were observed in the overall significance of models or their post-hoc comparisons with and without outliers removed. Also, given the large number of models, only those with significant effects are presented here; models with non-significant results are available on the tutorial GitHub page. Data duration was a significant predictor of: (a) microstate 1 (*F* = 5.83, corrected *p* = .004, η^2^ = 0.11), 2 (*F* = 13.50, corrected *p* < .001, η^2^ = 0.22), 4 (*F* = 21.10, corrected *p* < .001, η^2^ = 0.31), and 5 (*F* = 22.09, corrected *p* < .001, η^2^ = 0.32) GEV, (b) microstate 1 (*F* = 11.36, corrected *p* < .001, η^2^ = 0.19), 2 (*F* = 18.86, corrected *p* < .001, η^2^ = 0.29), 4 (*F* = 15.57, corrected *p* < .001, η^2^ = 0.25), and 5 (*F* = 22.26, corrected *p* < .001, η^2^ = 0.32) coverage, and (c) microstate 1 (*F* = 8.72, corrected *p* < .001, η^2^ = 0.16), 2 (*F* = 15.00, corrected *p* < .001, η^2^ = 0.24), 4 (*F* = 12.13, corrected *p* < .001, η^2^ = 0.21), and 5 (*F* = 19.71, corrected *p* < .001, η^2^ = 0.30) occurrence. Data duration was not a significant predictor of the duration of any microstate or the duration, GEV, coverage, and occurrence of microstate 3. Pairwise differences of data duration on temporal property values for the significant models are presented in Table 1, and their bias-corrected and accelerated 95% confidence intervals are provided in Supplementary Materials S11.

Given the large number of significant post-hoc comparisons, we present three additional exploratory analyses in Supplementary Materials S12 to aid in the interpretation of our results: (1) the Pearson correlation coefficient for each comparison showing high correlations between values even for statistically significant comparisons, and, at times, much higher correlations for significant comparisons than other non-significant comparisons, (2) the standard deviation of the paired difference scores for each comparison showing small standard deviations for some significant comparisons but comparatively large standard deviations for other non-significant comparisons, and (3) the coefficient of variation showing individual, within-participant variability in stability.

Data duration was not a significant predictor of the transition probability of any transition direction (see models available on tutorial GitHub page).

**Table 1:** Pairwise differences of data duration on temporal property values for significant models

	Data Duration Comparisons									
	1–2	1–3	1–4	1–5	2–3	2–4	2–5	3–4	3–5	4–5
M1 GEV	NS	NS	NS	NS	NS	NS	NS	**0.004** (0.001)	**0.005** (0.001)	NS
M2 GEV	NS	**0.008** (0.001)	NS	NS	**0.006** (0.001)	NS	NS	**-0.005** (0.001)	NS	NS
M4 GEV	**0.004** (0.001)	NS	**-0.006** (0.002)	NS	**-0.005** (001)	**-0.010** (0.001)	**-0.008** (0.002)	**-0.005** (0.001)	NS	NS
M5 GEV	NS	**0.006** (0.001)	**0.012** (0.002)	**0.007** (0.002)	**0.006** (0.001)	**0.013** (0.002)	**0.008** (0.002)	**0.006** (0.001)	NS	**-0.005** (0.001)
M1 Coverage	**-0.485** (0.125)	NS	NS	NS	NS	**0.741** (0.174)	**0.976** (0.211)	**0.645** (0.125)	**0.881** (0.174)	NS
M2 Coverage	**0.741** (0.146)	**1.034** (0.199)	NS	NS	NS	**-0.686** (0.199)	NS	**-0.978** (0.146)	**-0.664** (0.199)	NS
M4 Coverage	NS	NS	**-1.105** (0.221)	**-0.958** (0.254)	NS	**-1.222** (0.182)	**-1.075** (0.221)	**-0.971** (0.129)	**-0.824** (0.182)	NS
M5 Coverage	NS	NS	**1.452** (0.234)	NS	NS	**1.293** (0.194)	NS	**1.047** (0.139)	NS	**-0.759** (0.139)
M1 Occurrence	**-0.041** (0.012)	NS	NS	NS	NS	**0.071** (0.017)	**0.089** (0.021)	**0.056** (0.012)	**0.073** (0.017)	NS
M2 Occurrence	**0.070** (0.014)	**0.093** (0.017)	NS	NS	NS	**-0.068** (0.017)	NS	**-0.090** (0.014)	**-0.071** (0.017)	NS
M4 Occurrence	NS	NS	**-0.098** (0.019)	**-0.096** (0.022)	NS	**-0.098** (0.016)	**-0.096** (0.019)	**-0.074** (0.011)	**-0.071** (0.016)	NS
M5 Occurrence	NS	NS	**0.119** (0.021)	NS	NS	**0.093** (0.018)	NS	**0.093** (0.013)	NS	**-0.060** (0.013)

*Note.* M = microstate (e.g., M1 = microstate 1). Column headings represent data duration comparisons (e.g., 1–2 represents comparison between one- and two-minute data durations). Pairwise differences are presented as the **mean difference** (standard error). NS = non-significant.

Mean DSC values between neural source distributions of varying data durations across microstates are provided in Table 2. All mean DSC values were considered excellent (≥ 0.75). Sources from the five-minute data duration results displayed on an MRI brain are available on the tutorial GitHub page.

**Table 2:** Mean DSC values between neural source distributions of varying data durations across microstates

	1 min	2 min	3 min	4 min
1 min				
2 min	**0.81** (0.05); 0.71–87			
3 min	**0.79** (0.06); 0.70–89	**0.82** (0.04); 0.74-0.89		
4 min	**0.78** (0.08); 0.67-0.89	**0.82** (0.05); 0.73-0.91	**0.82** (0.09); 0.66-0.72	
5 min	**0.76** (0.05); 0.70-0.84	**0.80** (0.06); 0.72-0.91	**0.80** (0.06); 0.72-0.88	**0.82** (0.07); 0.73-0.91

*Note.* Presented as **mean** (standard deviation); range.

### Internal Consistency

**Fig. 2:**
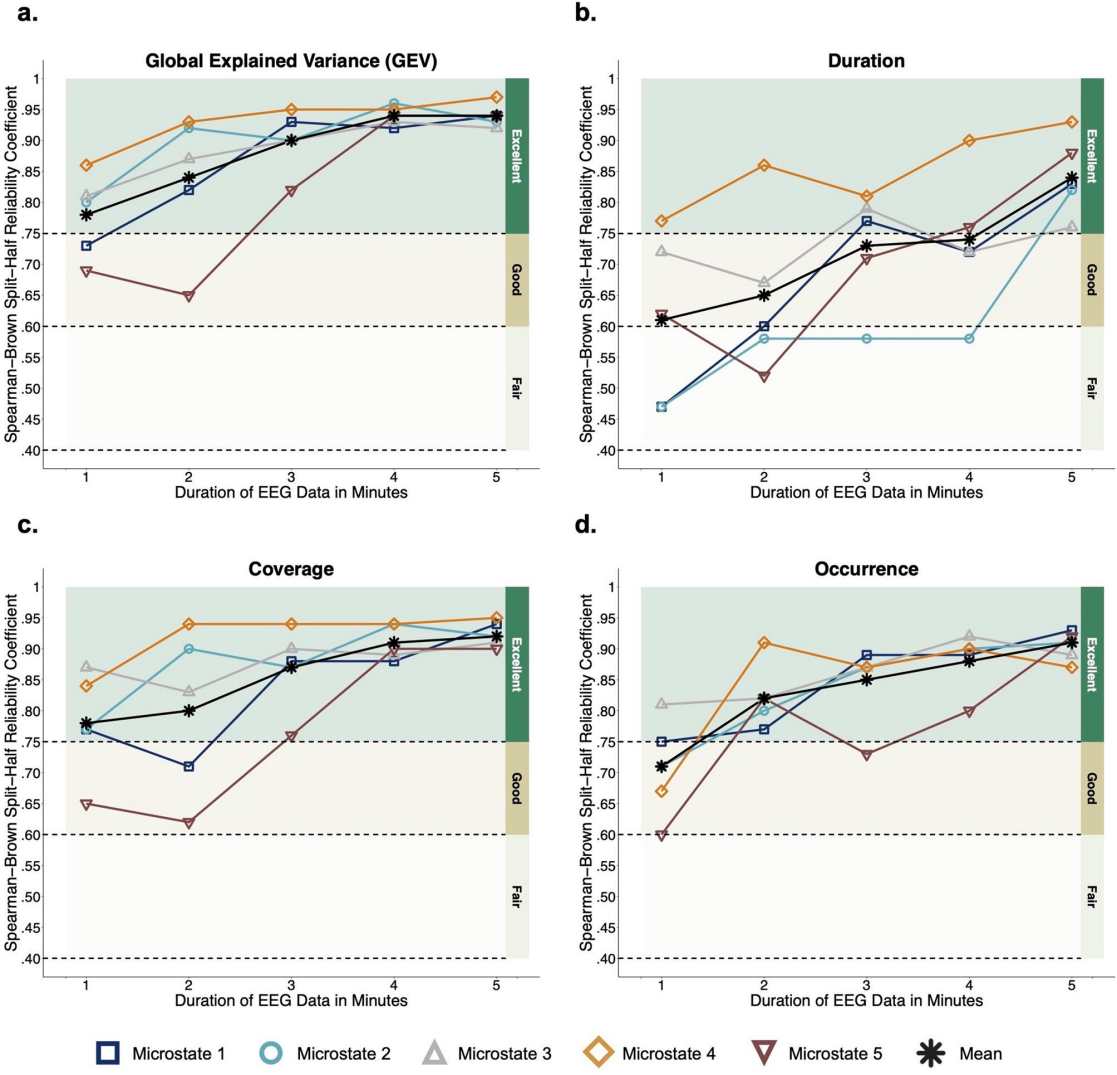
Split-half reliability of temporal properties

Results of internal consistency analyses are presented for models with outliers removed while models without outliers removed are available on the tutorial GitHub page. Spearman-Brown split-half reliability coefficients for microstate temporal properties are plotted in Fig. 2. Mean values for GEV across microstates were excellent for all data durations: one (M = 0.78; Range = 0.69-0.86), two (M = 0.84; Range = 0.65-0.93), three (M = 0.90; Range = 0.82-0.95), four (M = 0.94; Range = 0.92-0.96), and five (M = 0.94; Range = 0.92-0.97) minutes of data (Fig. 2a). Mean values for duration were good with one (M = 0.61; Range = 0.47-0.77), two (M = 0.65; Range = 0.52-0.86), three (M = 0.73; Range = 0.58-0.81), and four (M = 0.74; Range = 0.58-0.90) minutes of data, and excellent with five minutes of data (M = 0.84; Range = 0.76-0.93) (Fig. 2b). Mean values for coverage across microstates were excellent for all data durations: one (M = 0.78; Range = 0.65-0.87), two (M = 0.80; Range = 0.62-0.94), three (M = 0.87; Range = 0.76-0.94), four (M = 0.91; Range = 0.88-0.94), and five (M = 0.92; Range = 0.90-0.95) minutes of data (Fig. 2c). Mean values for occurrence across microstates were good with one minute of data (M = 0.71; Range = 0.60-0.81), and excellent with two (M = 0.82; Range = 0.77-0.91), three (M = 0.85; Range = 0.73-0.89), four (M = 0.88; Range = 0.80-0.92), and five (M = 0.91; Range = 0.87-0.93) minutes of data (Fig. 2d). Exact values for each microstate and data duration combination are available on the tutorial GitHub page. When outliers were not removed, the mean value for duration was in the fair rather than the good range at one minute, and in the excellent rather than the good range at three and four minutes; the only differences in mean values when comparing models with and without outliers removed (see models available on tutorial GitHub page).

**Fig. 3:**
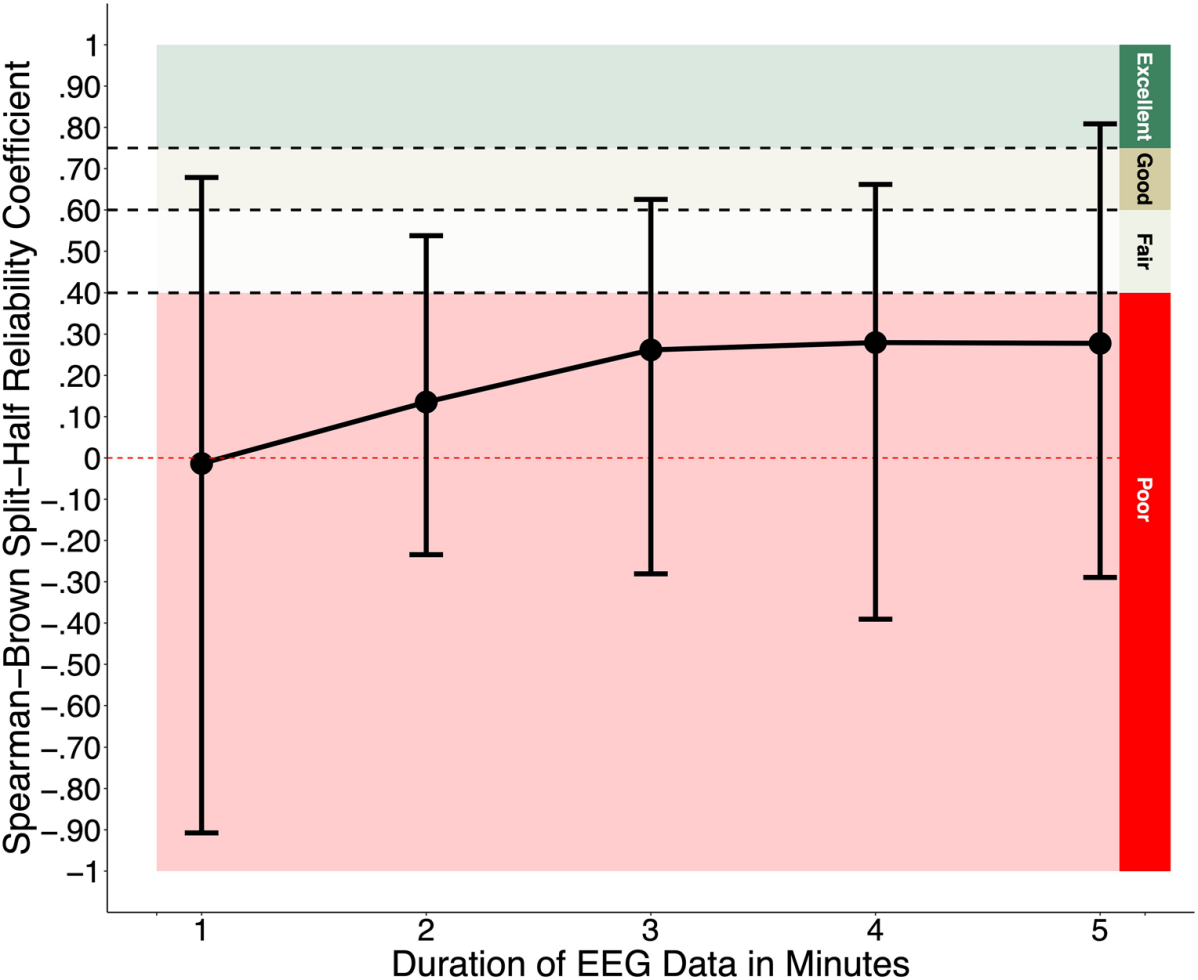
Split-half reliability of transition probabilities. *Note.* Bars indicate the minimum and maximum Spearman-Brown split-half reliability coefficient values for transition probabilities across transition directions for each data direction

The mean and range of Spearman-Brown split-half reliability coefficients for microstate transition probabilities across transition directions are plotted in Fig. 3 (outliers removed). No differences were observed between models with outliers removed and models without outlier removed (see models available on tutorial GitHub page). Mean values were poor for all data durations (one-minute M = 0.02; two-minute M = 0.16; three-minute M = 0.27; four-minute M = 0.27; five-minute M = 0.26), and the range of values indicated large variability across transition directions with the majority showing poor values (even anticorrelated) and a small handful showing fair, good, and excellent values (one-minute Range = -0.91-0.68; two-minute Range = -0.23-0.54; three-minute Range = -0.28-0.63; four-minute Range = -0.39-0.66; five-minute Range = -0.29-81). Exact values for each transition direction and data duration combination are available on the tutorial GitHub page.

**Fig. 4:**
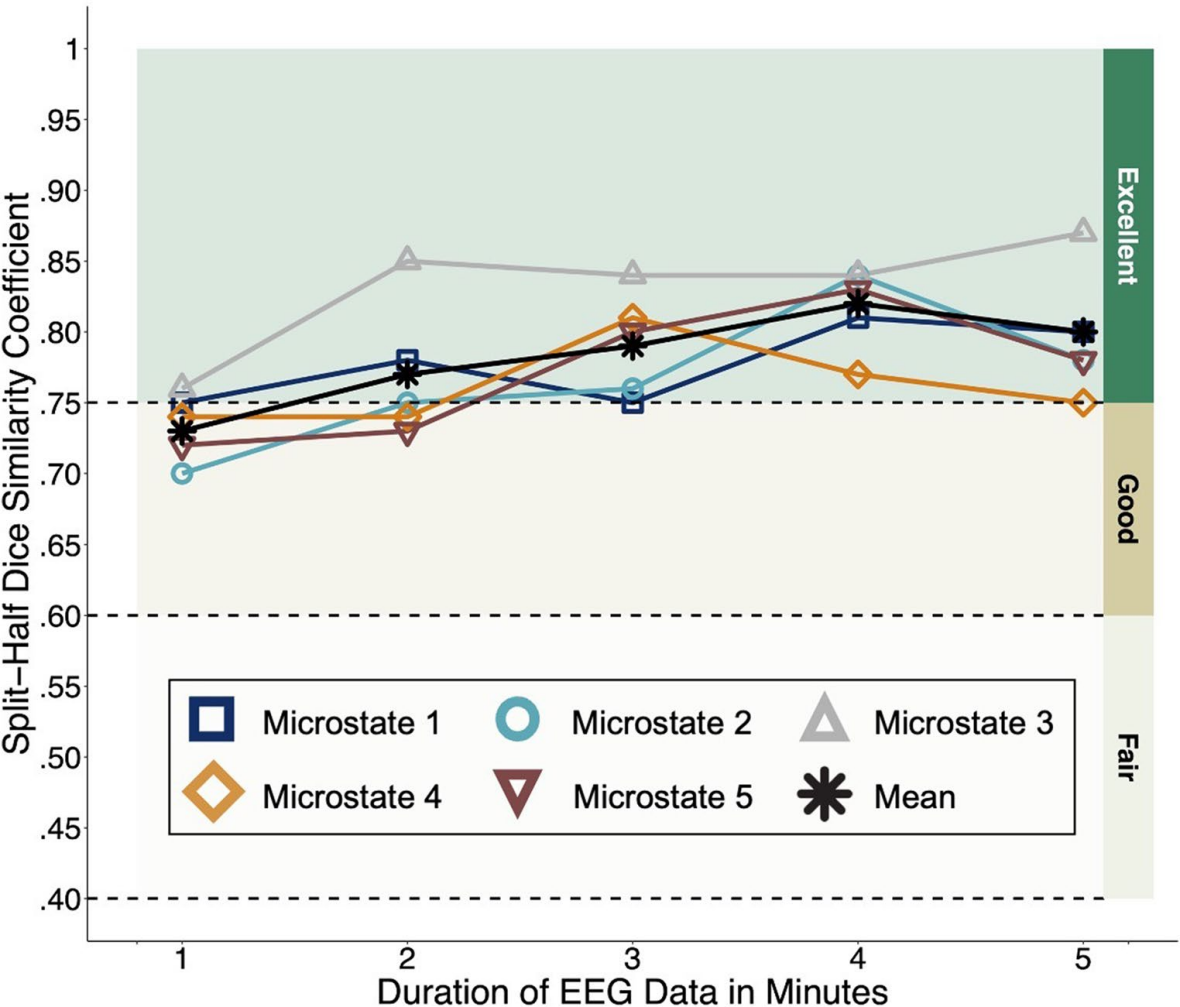
Split-half reliability of neural sources

Split-half DSC values for microstate sources are plotted in Fig. 4. Mean DSC values across microstates were good with one minute of data (M = 0.73; Range = 0.70-0.76) and excellent with two (M = 0.77; Range = 0.73-0.85), three (M = 0.79; Range = 0.75-0.84), four (M = 0.82; Range = 0.77-0.84), and five (M = 0.80; Range = 0.75-0.87) minutes of data. Exact values for each microstate and data duration combination are available on the tutorial GitHub page.

## Discussion

Reliably characterizing the functional properties of brain activity and organization during infancy provides a unique opportunity for understanding expected patterns of brain development and their association with developing infant abilities and early life experiences. EEG microstates are a promising but relatively unexplored method for measuring global patterns of brain activity and organization very early in development. As an important step in demonstrating the significant potential of using an EEG microstate-based analytic approach to study functional brain development, the current study examined the reliability of resting-state EEG microstates characterized during infancy across (i.e., group-level stability) and within (i.e., individual-level internal consistency) data of increasing durations. To further support wider availability and use of EEG microstate analysis for the study of brain development, a step-by-step tutorial for a free, easy-to-use, publicly available, and soon-to-be open-source EEG microstate analysis software package – Cartool – was also developed. And to facilitate open science practices, EEG data were shared in BIDS format on OpenNeuro. Findings revealed that video-watching resting-state EEG data collected from infants during the first year of life yielded highly reliable microstate-based measurements of brain function and organization with as little as one or four minutes of data, depending on the analytical goals. As a result, the current findings support the use of EEG microstates as a reliable and accessible analytical approach for studying the spatiotemporal dynamics of the developing brain from a very early age.

### Stability Across Data Durations

Group-level stability of microstate measures reflects the predictability of patterns observed when analyzing data from a group of individuals. It allows researchers to understand how much data and number of participants are required to develop a quantitative understanding of overall expected patterns of change in microstate measures with age. Findings indicated that across all data durations investigated (i.e., 1-5 min), five data-driven microstates explained the majority of topographic variance. Topographies derived from one minute of data were visually and quantitatively (i.e., spatial correlation) similar to those from two-, three-, four-, and five-minute data durations. Further, when topographic stability was assessed by varying sample size (i.e., *n* = 10-48), a minimum of 20 participants was sufficient for topographic stability with two minutes of data or more. Even with one minute of data, each sample size yielded the same five microstates, except for when the sample size was 30, which yielded six microstates. Importantly, topographies were also similar to those reported in previously published studies of microstates in children and adults (Bagdasarov et al. 2022, 2023 (4-8-year-olds); Custo et al. 2017 (6-87-year-olds); Hill et al. 2023 (4-12-year-olds); Michel and Koenig 2018 (review of primarily adult studies); Tomescu et al. 2018 (6-87-year-olds), indicating future potential for the direct comparison of their temporal properties, transition probabilities, and neural sources across the lifespan. This finding is especially promising for longitudinal work that aims to uncover individual differences in brain development and behavior from a very early age.

The stability of microstate temporal properties (GEV, duration, coverage, and occurrence) across data of increasing durations depended on the type of temporal property and microstate assessed. Overall, four patterns emerged: First, the temporal properties of microstate 3 were stable with just one minute of data. Microstate 3 had the highest values for all its temporal properties compared to the other microstates. Increased occurrence of microstate 3 compared to the other microstates may have contributed to earlier stability of its temporal properties (i.e., more instances for the computation of its reliability). Second, duration was stable with just one minute of data for all microstates. While GEV, coverage, and occurrence quantify how much microstates are represented in the EEG signal, duration quantifies how long microstates are present when they occur, regardless of how much they are represented in the EEG signal. Therefore, duration values may be less affected by the duration of the data being analyzed or, in other words, opportunities for its occurrence. Third, GEV, coverage, and occurrence were stable with four minutes of data for all microstates except microstate 5, which did not reach stability. Microstate 5 is highly spatially correlated with microstate 3. Indeed, prior work has shown that when only four microstates are used, microstate 3 becomes a combination of microstates 3 and 5 (Custo et al. 2017). Thus, it is possible for some instances that microstate 5 shows temporal property values similar to those of microstate 3 (i.e., when more spatially aligned with microstate 3). Additional research is needed to understand whether microstates 3 and 5 are functionally distinct during infancy.

The transition probabilities between microstates showed no differences in their values across data of increasing durations. However, this may be more reflective of their poor internal consistency rather than stability across different amounts of data, as discussed below.

Source distributions had good and excellent overlap (i.e., high DSC values) across data of increasing durations. Comparison of source distributions from varying data durations indicated stability of sources even with one minute of data. Taken together, the current results suggest that measurements of infant EEG microstate topographies are stable at the group-level with 20 participants and two minutes of data, that brain dynamics of the resulting topographies achieve stability with four or more minutes of data for most microstates, and that estimation of source distributions for each topography reach stability with as little as one minute of data.

### Internal Consistency

Internal consistency of microstate measures reflects the consistency and accuracy of measures at the individual-level. It allows researchers to understand how much data is required to reliably examine individual differences in brain (e.g., within-subject changes in microstate measures) and behavior (e.g., brain-behavior relationships). Similar to stability results, the internal consistency of microstate temporal properties within data of increasing durations depended on the type of temporal property and microstate assessed. On average, all temporal properties showed good or excellent internal consistency with just one minute of data, and excellent internal consistency with two or more minutes, except for duration which required five minutes to show excellent values. The slightly lower internal consistency of duration relative to other temporal properties may be due to the discontinuous nature of our preprocessed EEG data. During EEG preprocessing, data were segmented into one-second epochs and epochs were removed if they met artifact rejection criteria. Thus, each removed epoch may have altered the duration of a given microstate immediately preceding and/or following a removed epoch. For example, at the start or end of the epoch, a microstate’s duration may have been cut in half if the epoch before it or after it was removed during preprocessing. This would have less of an impact on GEV, coverage, and occurrence values, which are largely independent of duration. With more data (5 minutes), this disruption may have been averaged out.

Transition probabilities showed poor internal consistency for all data durations. There was also considerable variability in internal consistency values between transition directions, from highly negative to highly positive coefficients. Similar to the impact that preprocessing may have had on the internal consistency of duration at one minute of data, transition probabilities may have suffered from a similar problem, and potentially to a greater degree because the computation of each transition probability involved the activity of two microstates rather than one. However, in this case, as observed for duration, we would have expected an increase in internal consistency values with increasing data durations, which was not observed. Alternatively, transition probabilities may reflect state-related measures of brain activity that are modulated by environmental demands or external stimuli (e.g., idiosyncratic patterns of attention to movements of stimuli within the videos) while temporal properties may reflect trait-like measures of highly conserved patterns of brain activity and organization underlying more general cognitive (e.g., sustained attention) and/or sensory (e.g., processing of visual stimuli) domains. However, previous work in adults showed that transition probabilities did not distinguish between mental states (i.e., mind-wandering, verbalization, and visualization conditions; Antonova et al. 2022). The same study also indicated that compared to temporal properties, transition probabilities show much lower test-retest reliability (most ICCs falling below 0.7; Antonova et al. 2022), as did another study (most ICCs falling below 0.5; Kleinert et al. 2023); though neither study assessed internal consistency. Another hypothesis is that the infant brain does not have well-developed rules that govern intrinsic brain activity. Specifically, while microstate transitions are non-random in adults (Gschwind et al. 2015; Lehmann et al. 2005; Ville et al. 2010; Wackermann et al. 1993), they may be more spontaneous during infancy when the brain’s structural and functional organization is constantly and rapidly changing (Grayson and Fair 2017). Future longitudinal work is needed to elaborate on this possibility. Finally, first-order Markov models may not be appropriate for assessing microstate sequences, which may have contributed to their poor internal consistency. Previous work has demonstrated that microstate sequences are complex and show long-range dependencies; they cannot be adequately explained by simple first-order Markov models (Artoni et al. 2023; Ville et al. 2010). As such, higher-order dependencies and/or more sophisticated models need to be considered to accurately describe their syntax, which may in turn increase their reliability. Until further investigation, we do not recommend the use of first-order Markov transition probabilities with infant data.

The internal consistency of source distributions was good with one minute of data and excellent with two-, three-, four-, and five-minute data durations. This is line with previous research in adults indicating high reliability of resting-state EEG source localization with 1.5-2 minutes of data (Cannon et al. 2012) and suggests that the neural generators underlying specific microstates during infancy exhibit consistent and distinct spatial configurations. While these findings provide a key piece of data supporting each microstate as a unique neurobiological marker of brain function(s), the potential associations between microstate properties and specific aspects of behavior will require additional research.

Altogether, good or excellent internal consistency of temporal properties and source distributions was achieved with just one minute of data. Internal consistency was poor for transition probabilities at all data durations. Thus, to achieve a desirable level of internal consistency of infant EEG microstates, it is recommended that at least one minute of data is used for individual-level analyses. This will ensure that all microstate measures from individuals are consistent and dependable, allowing for valid conclusions to be drawn regarding their unique characteristics, within-subject changes, brain-behavior relationships, and developmental trajectories.

### Integration of Stability and Internal Consistency Findings

Our stability and internal consistency findings suggest that the amount of data required to achieve reliable estimates of microstate temporal properties varies based on the metric of interest. Indeed, we demonstrated that while microstate temporal properties reached high levels of internal consistency with one minute of data, in order to reach stability of these properties at least 4 minutes of data was required. Considering this, we worked to clarify how the stability of EEG microstate temporal properties changed across data of different durations through a series of supplemental analyses (Supplementary Materials S12). Briefly, we found that even when comparisons between data of different durations were statistically significant, the correlations between their values were very strong, and the standard deviations of their paired differences were smaller compared to when comparisons were not statistically significant. We also found large within-participant variability in how the temporal properties changed across data durations, with some participants showing stable values across data durations and other participants showing large differences. In addition to these supplemental analyses, we observed surprisingly small paired mean differences for statistically significant comparisons, including those between 4- and 5-minutes of data for microstate 5 (Table 1). In all, the results of our analyses suggest that statistical differences in the stability of temporal properties for most microstates are not present when four or more minutes of data are used to measure them. However, they also indicate that statistical findings of stability and internal consistency need to be interpreted within the specific context of their intended use. That is, although we used statistical significance to determine whether the temporal properties of microstates changed across data of different durations, statistical significance does not replace or imply biological significance. More specifically, the presence or absence of a statistically significant difference between the temporal properties of two data durations is agnostic as to whether their difference is biologically meaningful. As designed, the current study cannot inform this question (though see Supplementary Materials S12 for additional clarity). However, as in many areas of research, it does point to the importance of future research on how statistical significance can be used to inform measure selection and design when investigating biological processes and systems using EEG microstates.

Nevertheless, when considering the pragmatic implications of study results for future infant EEG microstate research, they indicate that four minutes of data are required to achieve both stable and internally consistent temporal properties for almost all microstates. This combination – stability and internal consistency – is likely to be critical for longitudinal analyses and those investigating individual differences in brain/behavior relationships and/or neurobiological mechanisms underlying the effects of discrete events (e.g., early intervention). Our results also suggest that while lacking stability, temporal properties derived from shorter durations of data are internally consistent and may still be useful in certain contexts. For example, if the goal is to investigate potential biomarkers with predictive utility, then one minute of data may be sufficient to identify microstate-related properties that indicate a potential outcome when a detailed understanding of mechanism is not required. In fact, resting-state EEG data are often collected as part of a larger battery of tasks, and, as a result, may be of brief duration. In this case, the use of this data to assess predictive utility and potentially suggest areas for further investigation into potential mechanisms is still possible. Nevertheless, regardless of use case, the current results suggest that the amount of data used to calculate EEG microstate properties should be identical across participants included in the same analyses.

Importantly, we did not perform supplemental analyses for transition probabilities because an acceptable level of internal consistency was not achieved. That is, internal consistency must be achieved to validly interpret the statistical findings of stability analyses. Therefore, despite stability reached at one minute according to statistical models, we view transition probabilities as measured in the current study as highly unreliably given their poor internal consistency.

### Strengths, Limitations, and Future Directions

The current study is the first to assess the psychometric reliability of EEG microstates – their topographies, temporal properties, transition probabilities, and neural sources – from infants during the first year of life. Our findings give guidance to researchers interested in using the microstate analytic approach in their own work; specifically, for how much data may be required for reliable results (i.e., approximately one or four minutes depending on the analysis goal). Given this guidance, and the unique advantages of this approach for investigating brain organization and functioning, microstates hold strong promise for individual differences research, which may lead to new insights characterizing the spatial and temporal dynamics of the infant brain and potential associations with emerging social, emotional, and cognitive skills. The current study is also the first to provide a comprehensive, step-by-step tutorial for performing microstate analysis in Cartool. Cartool does not require any computer programming knowledge (i.e., it is user-friendly) or software dependencies (i.e., it is a standalone program). As such, it makes performing microstate analysis feasible for researchers of varying computer programming skill levels. In addition, to support researchers who will want to perform source localization of infant microstates, MRI files (i.e., brain, head, grey matter, and segmented tissues) for 5-11-month-old infants are provided in the accompanying tutorial website.

This work has several limitations and avenues for future research. First, we did not have data from multiple sessions to assess the test-retest reliability of microstates. While previous work demonstrated adequate to excellent short- and long-term test-retest reliability of microstate measures over multiple sessions in adults (Antonova et al. 2022; Khanna et al. 2014; Kleinert et al. 2023; Liu et al. 2020; Popov et al. 2023), future work will need to directly assess this in infants. And while high reliability of microstate analysis during infancy opens the possibility of using this approach in longitudinal samples, reliability has not yet been systematically examined across childhood (e.g., toddlers, school-aged children, adolescents). This may be an important avenue for future research as previous work has indicated different levels of reliability of EEG metrics across developmental stages (Popov et al. 2023). Second, while EEG data was collected during video-watching to reduce movement-related artifacts, dynamic videos are not identical to traditional resting-state, and it is not clear whether these videos impacted microstate measures. Future work should assess whether different types of videos impact microstate measures differently. Also, video recordings of infants during EEG were not available to precisely assess whether infants were looking at the screen at all times. Third, we did not use individual MRI scans and EEG channel coordinate locations during source localization procedures, which have been previously demonstrated by Conte and Richards (2022) to reduce localization errors of infant event-related potentials. It will be critical for future research to discern whether individual MRI scans and EEG channel coordinate locations impact microstate source localization results or if template files are acceptable, which would increase the feasibility of performing source localization to researchers who do not have access to individual MRI scans and EEG channel coordinate location. Fourth, we did not directly examine stability past five minutes of data. Future research should elaborate on whether our statistically defined four-minute data cutoff is optimal for individual differences, mechanistic, and/or longitudinal work. Lastly, although the results of our investigation suggest that current methods for performing microstate analysis are compatible with EEG data collected from infants and yield reliable measures, future research is necessary to further understand the appropriateness of the current methods to facilitate longitudinal investigations of brain development at this age.

## Conclusion

In conclusion, we established microstate analysis as a feasible and reliable approach for characterizing infant brain development using continuous EEG. With this and our practical tutorial, developmental EEG researchers can begin exploring what new information about the developing brain during the first years of life can be gleaned using the microstate analytic approach. Given rapid changes in brain structure and function during infancy and early childhood (Bethlehem et al. 2022; Johnson 2001), we expect to see large changes in the temporal properties of microstates as resting-state networks reorganize and fine-tune across development. We hope microstate analysis can facilitate understanding of these changes by incorporating information across the entire brain, from all EEG channels, and in the source space.

### Electronic Supplementary Material

Below is the link to the electronic supplementary material.

Supplementary Material 1

## Data Availability

The data that support the findings of this study are openly available on OpenNeuro (OpenNeuro Accession Number: ds004635; https://openneuro.org/datasets/ds004635) and GitHub (https://github.com/gaffreylab/EEG-Microstate-Analysis-Tutorial).
