# Supplementary material for: Microstate Analysis of Continuous Infant EEG: Tutorial and Reliability
Source: Brain Topogr. 2024 Mar 2;37(4):496–513. doi: 10.1007/s10548-024-01043-5 (PMC11199263; doi:10.1007/s10548-024-01043-5)
Supplement: Supplementary file 1 — Supplementary Material 1 [file 10548_2024_1043_MOESM1_ESM.docx]

Supplementary Materials

Table of Contents

[Methods 2](#_Toc157029266)

[S1. Participant Recruitment and Eligibility 2](#_Toc157029267)

[S2. Participant Demographics 3](#_Toc157029268)

[S3. EEG Videos 4](#_Toc157029269)

[S4. EEG Data Quality Checks 5](#_Toc157029270)

[S5. Resampling Data During Microstate Analysis 6](#_Toc157029271)

[S6. Split-Half Segments 7](#_Toc157029272)

[Results 8](#_Toc157029273)

[S7. Descriptive Statistics of Microstate Temporal Properties and Transition Probabilities 8](#_Toc157029274)

[S8. Backfitting Details 10](#_Toc157029275)

[S9. Topographic Stability with Varying Data Durations and Number of Participants 11](#_Toc157029276)

[S10. Transition States 12](#_Toc157029277)

[S11. Confidence Intervals for Paired Mean Differences 13](#_Toc157029278)

[S12. Post-Hoc Exploratory Analyses 14](#_Toc157029279)

[Other 18](#_Toc157029280)

[S13. Software Versions 18](#_Toc157029281)

# Methods

## S1. Participant Recruitment and Eligibility

Participants were part of a larger study investigating how maternal experiences of bias and discrimination affect maternal mental and physical health during and after pregnancy and, subsequently, postnatal child development. Data collection for the parent study is in progress. The parent study is currently collecting questionnaire measures of discrimination, maternal and infant mental health, social connectedness, and child development from mothers during each trimester of pregnancy and when children are approximately 6-, 9-, and 24-months of age. At 6- and 9-months of age, mother-infant dyads participate in lab visits where resting-state EEG is recorded from infants and dyadic head-mounted eye-tracking during naturalistic play is recorded from infants and mothers. Although infants were to be seen at 6- and 9-months for EEG, some were seen at 5- or 7-months for their 6-month visit and at 7- or 10-months for their 9-month visit due to scheduling constraints or illness.

Participants were recruited through Project Hope 1000 (ClinicalTrials.gov Identifier: NCT04417283), social media posts and advertisements, the Research Participant at Duke Database maintained by the Human Subjects Coordinator for the Department of Psychology and Neuroscience at Duke, the Duke Behavioral Research participant pool, and the Perinatal Psychiatry Behavioral Health Team directed by Dr. Andrea Diaz Stransky. Inclusion criteria included maternal age of at least 18 years at time of consent, ability to understand English, and singleton gestation less than 24 weeks. Exclusionary criteria included non-viable pregnancy, maternal medical condition in the opinion of investigators that would place the mother at unacceptable risk of injury or confound data interpretation, infant congenital, genetic, or neurologic disorder, and infant birth before 34 weeks of gestation.

At the time of data analyses for the current manuscript, EEG data from 71 infants was recorded. Infants had EEG data at either their 6-month or 9-month visits, or at both visits, resulting in 107 sessions from 65 infants. Sessions that did not result in at least five minutes of clean, artifact-free data after preprocessing were removed, resulting in 72 EEG sessions from 48 infants. In order for each infant to be represented only once in the final data set, for each infant that had EEG at both their 6- and 9-month visits, we kept the EEG recording that was closest in date to when the infant was exactly 6- or 9-months-old. After removing 24 EEG sessions, each of the 48 infants had one EEG session at either their 6- or 9-month visit. The final set of EEG sessions from the 48 infants were collected between February 24, 2022 and October 19, 2023.

## S2. Participant Demographics

|  | Mean (SD) | Range |
| --- | --- | --- |
| Age (months) | 8.01 (1.56) | 5.98 – 10.42 |
|  |  |  |
|  | *n* | Percent (%) |
| Biological Sex |  |  |
| Males | 27 | 56.25% |
| Females | 21 | 43.75% |
| Race |  |  |
| White | 36 | 75.00% |
| Multiracial | 7 | 14.58% |
| Black or African American | 3 | 6.25% |
| Asian | 1 | 2.08% |
| Other | 1 | 2.08% |
| Native Hawaiian or Other Pacific Islander | 0 | 0% |
| Ethnicity |  |  |
| Not Hispanic or Latino | 42 | 87.50% |
| Hispanic or Latino | 6 | 12.50% |
| Maternal Education |  |  |
| Some Grade School | 0 | 0% |
| Completed Grade School | 0 | 0% |
| Some High School | 1 | 2.08% |
| High School Diploma | 1 | 2.08% |
| Some College or 2-Year Degree | 1 | 2.08% |
| 4-Year College Degree | 16 | 33.33% |
| Some School Beyond College | 1 | 2.08% |
| Professional or Graduate Degree | 28 | 58.33% |

*Note.* All demographic variables were obtained at the time of study enrollment, except for age, which represents infants’ ages at the time of their EEG session.

## S3. EEG Videos

Dynamic videos were purchased from <https://wayokids.com/>. 90-second snippets from their *Hey Bear Sensory* collection were used. Snippets were selected to encourage visual engagement and minimize motion through presentation of bright and sharp images with sound. Selected snippets did not contain elaborate storylines. For example, videos included a dancing moon and dancing stars, a turtle swimming, leaves appearing one-by-one on a tree, the formation of a rainbow, etc. These videos are often used by parents during bedtime to encourage sleep. As such, they are thought to be relaxing. Due to copyright issues, the exact videos used are not included here, but samples are available on YouTube at the following links. Additional information is available upon request.

1) <https://www.youtube.com/watch?v=UyANUnGoGcE>

2) <https://www.youtube.com/watch?v=qCMZYEIlztY>

3) <https://www.youtube.com/watch?v=DsexoBXPedA>

## S4. EEG Data Quality Checks

Participants were only included in analyses if they met quality checks.

Criteria included:

1. Bad Channels
   - Number of bad channels must not exceed 15 (approximately 15% of 105 total channels).
   - Visualization of bad channels must not show clusters of bad channels.
2. Artifact Subspace Reconstruction (ASR)
   - Data length after ASR must exceed 60 seconds.
3. Independent Component Analysis (ICA)
   - Visualization of decomposition must appear “normal” and “appropriate.”
   - Visualization of flagged components must appear artifact-related.
   - Retained variance after removal of flagged components must exceed 50%.
4. Channel Power Spectra
   - Visualization of channel power spectra must appear “normal” and “appropriate.”
5. Data Duration
   - At least five minutes or 300 seconds of clean, artifact-free data must be available.

Descriptive statistics of resting-state EEG data quality metrics for *n* = 48 sample of infants.

|  | Number of Channels Removed and Interpolated | File Length in Seconds After ASR | Number of Independent Components Rejected | Percent Variance Retained After Removal of Independent Components | File Length in Seconds After Preprocessing |
| --- | --- | --- | --- | --- | --- |
| Mean | 4.52 | 497.30 | 4.46 | 88.33 | 461.29 |
| SD | 2.43 | 127.90 | 2.15 | 5.39 | 119.06 |
| Minimum | 0 | 294.72 | 1 | 74.77 | 301 |
| Maximum | 9 | 787.89 | 9 | 98.29 | 752 |

*Note.* ASR = Artifact Subspace Reconstruction. SD = standard deviation.

## S5. Resampling Data During Microstate Analysis

Number of Subsamples Entered in Cartool

Stage 1:

- 1 Minute: 50 Epochs, of 2000[TF], Covering 99.9% Data
- 2 Minutes: 50 Epochs, of 4000[TF], Covering 99.9% Data
- 3 Minutes: 50 Epochs, of 6000[TF], Covering 99.9% Data
- 4 Minutes: 50 Epochs, of 8000[TF], Covering 99.9% Data
- 5 Minutes: 50 Epochs, of 10000[TF], Covering 99.9% Data

Stage 2:

- 1 Minute: 100 Epochs, of 800[TF], Covering 99.7% Data
- 2 Minutes: 100 Epochs, of 800[TF], Covering 99.9% Data
- 3 Minutes: 100 Epochs, of 800[TF], Covering 99.9% Data
- 4 Minutes: 100 Epochs, of 800[TF], Covering 99.9% Data
- 5 Minutes: 100 Epochs, of 800[TF], Covering 99.9% Data

## S6. Split-Half Segments

|  | Segments and Range of Time Frames they Represent | | | | | |
| --- | --- | --- | --- | --- | --- | --- |
| Data Duration | 1 | 2 | 3 | 4 | 5 | 6 |
| 1 Minute | 0-2499 | 2500-4999 | 5000-7499 | 7500-9999 | 10000-12499 | 12500-14999 |
| 2 Minutes | 0-4999 | 5000-9999 | 10000-14999 | 15000-19999 | 20000-24999 | 25000-29999 |
| 3 Minutes | 0-7499 | 7500-14999 | 15000-22499 | 22500-29999 | 30000-37499 | 37500-44999 |
| 4 Minutes | 0-9999 | 10000-19999 | 20000-29999 | 30000-39999 | 40000-49999 | 50000-59999 |
| 5 Minutes | 0-12499 | 12500-24999 | 25000-37499 | 37500-49999 | 50000-62499 | 62500-74999 |

* Each time frame represents 4 ms of data.

Equal Segments

- One-minute: Six, 10-second segments
- Two-minutes: Six, 20-second segments
- Three-minutes: Six, 30-second segments
- Four-minutes: Six, 40-second segments
- Five-minutes: Six, 50-second segments

Even segments (2, 4, 6) were combined, and odd segments (1, 3, 5) were combined, resulting in two files for each participant.

# Results

## S7. Descriptive Statistics of Microstate Temporal Properties and Transition Probabilities

**GEV**

|  | Microstate 1 | Microstate 2 | Microstate 3 | Microstate 4 | Microstate 5 |
| --- | --- | --- | --- | --- | --- |
| 1 Minute | **.07** (.02) | **.09** (.03) | **.27** (.05) | **.08** (.03) | **.09** (.02) |
| 2 Minutes | **.07** (.02) | **.09** (.03) | **.27** (.05) | **.07** (.03) | **.09** (.02) |
| 3 Minutes | **.08** (.02) | **.08** (.02) | **.27** (.04) | **.08** (.03) | **.08** (.02) |
| 4 Minutes | **.07** (.02) | **.08** (.02) | **.27** (.04) | **.08** (.03) | **.07** (.02) |
| 5 Minutes | **.07** (.02) | **.08** (.02) | **.26** (.04) | **.08** (.03) | **.08** (.02) |

*Note.* Presented as **mean** (standard deviation).

**Duration** (milliseconds)

|  | Microstate 1 | Microstate 2 | Microstate 3 | Microstate 4 | Microstate 5 |
| --- | --- | --- | --- | --- | --- |
| 1 Minute | **78.83**(5.12) | **80.62** (5.50) | **101.02** (8.46) | **83.01** (9.16) | **83.43** (6.01) |
| 2 Minutes | **78.76** (4.60) | **79.96** (5.46) | **102.08** (6.76) | **82.44** (7.68) | **83.50** (4.79) |
| 3 Minutes | **79.10** (4.24) | **79.77** (4.34) | **102.66** (6.41) | **82.50** (6.70) | **82.30** (4.54) |
| 4 Minutes | **78.55** (4.21) | **80.30** (4.01) | **102.35** (5.63) | **83.79** (6.51) | **81.76** (4.49) |
| 5 Minutes | **78.19** (3.79) | **79.86** (4.19) | **101.91** (5.00) | **83.30** (6.16) | **82.48** (4.64) |

*Note.* Presented as **mean** (standard deviation).

**Coverage** (percentage of time)

|  | Microstate 1 | Microstate 2 | Microstate 3 | Microstate 4 | Microstate 5 |
| --- | --- | --- | --- | --- | --- |
| 1 Minute | **16.14** (2.97) | **17.63** (3.82) | **32.89** (4.21) | **16.12** (4.26) | **17.22** (2.78) |
| 2 Minutes | **16.63** (2.71) | **16.88** (3.39) | **33.43** (3.92) | **16.00** (3.92) | **17.06** (2.80) |
| 3 Minutes | **16.53** (2.77) | **16.59** (3.14) | **33.81** (3.51) | **16.25** (3.79) | **16.81** (2.70) |
| 4 Minutes | **15.89** (2.74) | **17.57** (3.12) | **33.55** (3.29) | **17.22** (3.75) | **15.77** (2.79) |
| 5 Minutes | **15.65** (2.67) | **17.26** (3.13) | **33.49** (3.13) | **17.08** (3.65) | **16.53** (2.76) |

*Note.* Presented as **mean** (standard deviation).

**Occurrence** (frequency per second)

|  | Microstate 1 | Microstate 2 | Microstate 3 | Microstate 4 | Microstate 5 |
| --- | --- | --- | --- | --- | --- |
| 1 Minute | **1.81** (0.27) | **1.92** (0.33) | **2.73** (0.26) | **1.68** (0.31) | **1.81** (0.25) |
| 2 Minutes | **1.85** (0.23) | **1.85** (0.27) | **2.74** (0.24) | **1.68** (0.29) | **1.79** (0.25) |
| 3 Minutes | **1.84** (0.23) | **1.82** (0.27) | **2.76** (0.22) | **1.70** (0.28) | **1.79** (0.24) |
| 4 Minutes | **1.78** (0.23) | **1.91** (0.26) | **2.75** (0.21) | **1.78** (0.27) | **1.69** (0.24) |
| 5 Minutes | **1.77** (0.23) | **1.89** (0.26) | **2.76** (0.20) | **1.78** (0.27) | **1.75** (0.24) |

*Note.* Presented as **mean** (standard deviation).

**Transition Probabilities**

|  | From1-To2 | From1-To3 | From1-To4 | From1-To5 |
| --- | --- | --- | --- | --- |
| 1 Minute | 1.03 (0.22) | 1.10 (0.14) | 0.91 (0.20) | 0.88 (0.15) |
| 2 Minutes | 1.03 (0.16) | 1.14 (0.11) | 0.90 (0.14) | 0.87 (0.13) |
| 3 Minutes | 1.03 (0.11) | 1.14 (0.09) | 0.90 (0.13) | 0.88 (0.11) |
| 4 Minutes | 1.01 (0.10) | 1.13 (0.09) | 0.91 (0.11) | 0.87 (0.10) |
| 5 Minutes | 1.01 (0.09) | 1.13 (0.08) | 0.90 (0.09) | 0.88 (0.09) |

*Note.* Presented as **mean** (standard deviation).

|  | From2-To1 | From2-To3 | From2-To4 | From2-To5 |
| --- | --- | --- | --- | --- |
| 1 Minute | 0.96 (0.17) | 1.16 (0.14) | 0.89 (0.19) | 0.90 (0.20) |
| 2 Minutes | 0.99 (0.14) | 1.15 (0.11) | 0.86 (0.13) | 0.92 (0.16) |
| 3 Minutes | 0.97 (0.11) | 1.18 (0.10) | 0.85 (0.13) | 0.91 (0.16) |
| 4 Minutes | 0.99 (0.11) | 1.16 (0.08) | 0.88 (0.10) | 0.89 (0.12) |
| 5 Minutes | 1.00 (0.10) | 1.17 (0.07) | 0.87 (0.09) | 0.88 (0.11) |

*Note.* Presented as **mean** (standard deviation).

|  | From3-To1 | From3-To2 | From3-To4 | From3-To5 |
| --- | --- | --- | --- | --- |
| 1 Minute | 1.03 (0.15) | 1.03 (0.15) | 0.95 (0.17) | 1.02 (0.12) |
| 2 Minutes | 1.03 (0.10) | 1.04 (0.10) | 0.95 (0.11) | 1.01 (0.11) |
| 3 Minutes | 1.04 (0.09) | 1.02 (0.07) | 0.94 (0.10) | 1.02 (0.10) |
| 4 Minutes | 1.02 (0.07) | 1.04 (0.07) | 0.97 (0.09) | 1.00 (0.09) |
| 5 Minutes | 1.02 (0.07) | 1.04 (0.07) | 0.96 (0.07) | 1.02 (0.08) |

*Note.* Presented as **mean** (standard deviation).

|  | From4-To1 | From4-To2 | From4-To3 | From4-To5 |
| --- | --- | --- | --- | --- |
| 1 Minute | 0.90 (0.19) | 0.89 (0.18) | 1.14 (0.17) | 1.13 (0.17) |
| 2 Minutes | 0.92 (0.14) | 0.86 (0.16) | 1.15 (0.13) | 1.11 (0.15) |
| 3 Minutes | 0.90 (0.11) | 0.90 (0.12) | 1.13 (0.11) | 1.10 (0.14) |
| 4 Minutes | 0.91 (0.10) | 0.90 (0.09) | 1.13 (0.09) | 1.10 (0.14) |
| 5 Minutes | 0.90 (0.10) | 0.89 (0.09) | 1.14 (0.09) | 1.09 (0.12) |

*Note.* Presented as **mean** (standard deviation).

|  | From5-To1 | From5-To2 | From5-To3 | From5-To4 |
| --- | --- | --- | --- | --- |
| 1 Minute | 0.89 (0.16) | 0.94 (0.19) | 1.06 (0.15) | 1.12 (0.21) |
| 2 Minutes | 0.91 (0.13) | 0.89 (0.14) | 1.07 (0.12) | 1.17 (0.17) |
| 3 Minutes | 0.90 (0.10) | 0.90 (0.12) | 1.06 (0.08) | 1.20 (0.18) |
| 4 Minutes | 0.88 (0.10) | 0.92 (0.12) | 1.06 (0.10) | 1.20 (0.16) |
| 5 Minutes | 0.87 (0.09) | 0.92 (0.09) | 1.06 (0.08) | 1.19 (0.15) |

*Note.* Presented as **mean** (standard deviation).

## S8. Backfitting Details

Time frames that did not meet the minimum spatial correlation threshold (.50) during backfitting for each data duration:

- 1 Minute 🡪 6.45%
- 2 Minutes 🡪 7.00%
- 3 Minutes 🡪 7.28%
- 4 Minutes 🡪 7.49%
- 5 Minutes 🡪 7.70%

These time frames were thus unlabeled during backfitting.

Descriptive Statistics for Global Field Power (GFP):

|  | Microstate 1 | Microstate 2 | Microstate 3 | Microstate 4 | Microstate 5 |
| --- | --- | --- | --- | --- | --- |
| 1 Minute | 1.11 (0.08) | 1.14 (0.08) | 1.32 (0.08) | 1.13 (0.05) | 1.14 (0.06) |
| 2 Minutes | 1.11 (0.06) | 1.14 (0.07) | 1.31 (0.06) | 1.12 (0.05) | 1.15 (0.05) |
| 3 Minutes | 1.12 (0.06) | 1.13 (0.06) | 1.31 (0.05) | 1.13 (0.05) | 1.13 (0.04) |
| 4 Minutes | 1.12 (0.06) | 1.14 (0.05) | 1.31 (0.05) | 1.14 (0.05) | 1.13 (0.04) |
| 5 Minutes | 1.11 (0.05) | 1.14 (0.05) | 1.31 (0.05) | 1.14 (0.05) | 1.14 (0.05) |

*Note.* Presented as **mean** (standard deviation).

Descriptive Statistics for Spatial Correlation:

|  | Microstate 1 | Microstate 2 | Microstate 3 | Microstate 4 | Microstate 5 |
| --- | --- | --- | --- | --- | --- |
| 1 Minute | .66 (.02) | .67 (.02) | .74 (.02) | .67 (.02) | .68 (.02) |
| 2 Minutes | .66 (.02) | .67 (.02) | .74 (.02) | .66 (.02) | .68 (.02) |
| 3 Minutes | .66 (.02) | .67 (.02) | .73 (.02) | .67 (.02) | .67 (.01) |
| 4 Minutes | .66 (.02) | .66 (.02) | .73 (.02) | .67 (.02) | .67 (.01) |
| 5 Minutes | .66 (.02) | .66 (.02) | .73 (.02) | .66 (.01) | .67 (.01) |

*Note.* Presented as **mean** (standard deviation).

## S9. Topographic Stability with Varying Data Durations and Number of Participants

Participants were randomly assigned a number between 1 and 48 and reordered from smallest to largest based on their number. For each data duration, microstate analyses (stages 1 and 2 only) were performed separately for participants 1-10, 1-20, 1-30, and 1-48. The meta-criterion informed the optimal number of microstates for each. Topographies are available in a PDF file on <https://github.com/gaffreylab/EEG-Microstate-Analysis-Tutorial/>

## S10. Transition States

Below is an example of a seven-microstate solution for the five-minute data duration. The first microstate represents a transition state. Topographically, it represents the transition between two canonical microstates, and it is spatially poorly correlated with any of the canonical microstates that have been observed in prior work. Thus, the seven-microstate solution should be excluded from being a candidate of the optimal solution for the five-minute data duration, regardless of its meta-criterion value. While this transition state may be meaningful (i.e., potentially reflecting a common transition between two specific microstates), its interpretation is unclear in the context of the available literature.

## S11. Confidence Intervals for Paired Mean Differences

Bias-corrected and accelerated 95% confidence intervals of the paired mean differences for significant post-hoc comparisons, calculated by performing nonparametric bootstrap resampling (5000 resamples).

|  | Data Duration Comparisons | | | | | | | | | |
| --- | --- | --- | --- | --- | --- | --- | --- | --- | --- | --- |
|  | 1-2 | 1-3 | 1-4 | 1-5 | 2-3 | 2-4 | 2-5 | 3-4 | 3-5 | 4-5 |
| M1 GEV | NS | NS | NS | NS | NS | NS | NS | -.004; .011 | -.002; .013 | NS |
| M2 GEV | NS | -.003; .018 | NS | NS | -.004; .016 | NS | NS | -.014; .004 | NS | NS |
| M4 GEV | -.007; .016 | NS | -.017; .006 | NS | -.015; .005 | -.021; .0004 | -.018; .002 | -.015; .005 | NS | NS |
| M5 GEV | NS | -.001; .013 | .005; .019 | .0001; .014 | -.0004; .013 | .006; .020 | .0008; .015 | -.0001; .013 | NS | -.011; .001 |
| M1 Coverage | -1.650; 0.639 | NS | NS | NS | NS | -0.383; 1.880 | -0.122; 2.080 | -0.503; 1.760 | -0.255; 1.980 | NS |
| M2 Coverage | -0.698; 2.150 | -0.401; 2.420 | NS | NS | NS | -2.030; 0.566 | NS | -2.280; 0.190 | -1.970; 0.532 | NS |
| M4 Coverage | NS | NS | -2.680; 0.532 | -2.490; 0.662 | NS | -2.760; 0.305 | -2.540; 0.451 | -2.440; 0.541 | -2.260; 0.659 | NS |
| M5 Coverage | NS | NS | 0.229; 2.440 | NS | NS | 0.126; 2.310 | NS | -0.094; 2.070 | NS | -1.820; 0.323 |
| M1 Occurrence | -0.140; 0.060 | NS | NS | NS | NS | -0.022; 0.163 | -0.006; 0.178 | -0.040; 0.147 | -0.024; 0.163 | NS |
| M2 Occurrence | -0.050; 0.191 | -0.026; 0.214 | NS | NS | NS | -0.178; 0.033 | NS | -0.203; 0.010 | -0.184; 0.031 | NS |
| M4 Occurrence | NS | NS | -0.208; 0.019 | -0.202; 0.020 | NS | -0.207; 0.015 | -0.205; 0.015 | -0.181; 0.037 | -0.178; 0.038 | NS |
| M5 Occurrence | NS | NS | 0.025; 0.215 | NS | NS | -0.003; 0.190 | NS | -0.003; 0.187 | NS | -0.156; 0.033 |

## S12. Post-Hoc Exploratory Analyses

Note: Outlier detection was not performed for post-hoc analyses since they were exploratory and main results did not change when outliers were or were not included in models.

**1. Correlations between values of each comparison**

|  | Data Duration Comparisons | | | | | | | | | |
| --- | --- | --- | --- | --- | --- | --- | --- | --- | --- | --- |
|  | 1-2 | 1-3 | 1-4 | 1-5 | 2-3 | 2-4 | 2-5 | 3-4 | 3-5 | 4-5 |
| M1 GEV | .88 | .84 | .82 | .80 | .96 | .93 | .92 | .98* | .96* | .98 |
| M2 GEV | .93 | .93* | .90 | .90 | .96* | .95 | .95 | .97* | .97 | .99 |
| M3 GEV | .92 | .89 | .88 | .84 | .96 | .93 | .91 | .98 | .96 | .98 |
| M4 GEV | .94* | .93 | .91* | .90 | .98* | .96* | .95* | .99* | .98 | .99 |
| M5 GEV | .85 | .78* | .71* | .68* | .95* | .87* | .85* | .94* | .93 | .98* |
| M1 Duration | .85 | .77 | .72 | .66 | .89 | .85 | .78 | .93 | .89 | .95 |
| M2 Duration | .87 | .80 | .77 | .78 | .91 | .89 | .88 | .90 | .92 | .95 |
| M3 Duration | .88 | .81 | .85 | .81 | .91 | .91 | .84 | .95 | .91 | .96 |
| M4 Duration | .91 | .87 | .83 | .82 | .94 | .90 | .89 | .96 | .97 | .99 |
| M5 Duration | .76 | .76 | .69 | .74 | .87 | .80 | .80 | .90 | .90 | .96 |
| M1 Coverage | .91* | .87 | .83 | .81 | .95 | .91* | .89* | .97* | .96* | .99 |
| M2 Coverage | .92* | .90* | .87 | .86 | .96 | .94* | .94 | .97* | .97* | .99 |
| M3 Coverage | .90 | .88 | .88 | .86 | .95 | .93 | .91 | .98 | .96 | .98 |
| M4 Coverage | .94 | .93 | .91* | .91* | .98 | .96* | .96* | .99* | .98* | .99 |
| M5 Coverage | .87 | .80 | .74* | .70 | .95 | .89* | .86 | .95* | .93 | .98* |
| M1 Occurrence | .89* | .83 | .81 | .78 | .94 | .90* | .88* | .96* | .94* | .98 |
| M2 Occurrence | .92* | .91* | .90 | .88 | .95 | .93* | .93 | .94* | .95* | .98 |
| M3 Occurrence | .89 | .88 | .86 | .86 | .90 | .88 | .88 | .98 | .97 | .98 |
| M4 Occurrence | .92 | .90 | .89* | .87* | .97 | .96* | .93* | .99* | .97* | .99 |
| M5 Occurrence | .87 | .80 | .76* | .71 | .94 | .89* | .86 | .95* | .94 | .98* |

*Note.* * = statistically significant comparison.

**2. Standard deviations of the paired difference scores of each comparison**

|  | Data Duration Comparisons | | | | | | | | | |
| --- | --- | --- | --- | --- | --- | --- | --- | --- | --- | --- |
|  | 1-2 | 1-3 | 1-4 | 1-5 | 2-3 | 2-4 | 2-5 | 3-4 | 3-5 | 4-5 |
| M1 GEV | .010 | .011 | .012 | .012 | .005 | .007 | .008 | .004* | .005* | .003 |
| M2 GEV | .010 | .011* | .013 | .013 | .007* | .008 | .009 | .006* | .005 | .003 |
| M3 GEV | .020 | .023 | .024 | .027 | .013 | .017 | .019 | .008 | .012 | .007 |
| M4 GEV | .011* | .012 | .013* | .013 | .005* | .007* | .008* | .004* | .005 | .003 |
| M5 GEV | .012 | .013* | .015* | .016* | .007* | .010* | .011* | .006* | .007 | .003* |
| M1 Duration | 2.746 | 3.287 | 3.622 | 3.857 | 2.146 | 2.473 | 2.876 | 1.585 | 1.971 | 1.362 |
| M2 Duration | 2.828 | 3.301 | 3.510 | 3.463 | 2.371 | 2.622 | 2.624 | 1.898 | 1.759 | 1.369 |
| M3 Duration | 4.126 | 4.975 | 4.753 | 5.288 | 2.843 | 2.880 | 3.705 | 2.095 | 2.729 | 1.675 |
| M4 Duration | 3.908 | 4.685 | 5.252 | 5.432 | 2.751 | 3.419 | 3.544 | 1.759 | 1.692 | 1.115 |
| M5 Duration | 3.902 | 3.888 | 4.346 | 4.044 | 2.347 | 2.937 | 2.970 | 2.013 | 2.024 | 1.327 |
| M1 Coverage | 1.260* | 1.500 | 1.667 | 1.760 | 0.847 | 1.139* | 1.240* | 0.679* | 0.819* | 0.465 |
| M2 Coverage | 1.464* | 1.711* | 1.911 | 1.961 | 0.992 | 1.136* | 1.165 | 0.823* | 0.764* | 0.497 |
| M3 Coverage | 1.828 | 1.980 | 2.017 | 2.208 | 1.200 | 1.518 | 1.655 | 0.725 | 0.981 | 0.670 |
| M4 Coverage | 1.450 | 1.581 | 1.776* | 1.824* | 0.782 | 1.058* | 1.164* | 0.570* | 0.708* | 0.410 |
| M5 Coverage | 1.396 | 1.730 | 1.996* | 2.137 | 0.852 | 1.339* | 1.470 | 0.877* | 0.990 | 0.517* |
| M1 Occurrence | 0.127* | 0.154 | 0.161 | 0.172 | 0.081 | 0.101* | 0.112* | 0.065* | 0.080* | 0.047 |
| M2 Occurrence | 0.135* | 0.138* | 0.149 | 0.160 | 0.088 | 0.100* | 0.102 | 0.092* | 0.082* | 0.054 |
| M3 Occurrence | 0.119 | 0.126 | 0.133 | 0.136 | 0.108 | 0.114 | 0.114 | 0.050 | 0.058 | 0.039 |
| M4 Occurrence | 0.122 | 0.135 | 0.139* | 0.153* | 0.073 | 0.083* | 0.108* | 0.046* | 0.067* | 0.046 |
| M5 Occurrence | 0.125 | 0.153 | 0.169* | 0.185 | 0.082 | 0.114* | 0.127 | 0.076* | 0.085 | 0.054* |

*Note.* * = statistically significant comparison.

**3. Within-subject coefficient of variation**

|  | **Mean** | **Standard Deviation** | **Min** | **Max** |
| --- | --- | --- | --- | --- |
| **GEV** |  |  |  |  |
| Microstate 1 | 7.70 | 3.26 | 3.39 | 19.40 |
| Microstate 2 | 7.49 | 2.70 | 2.60 | 13.58 |
| Microstate 3 | 4.40 | 2.28 | 0.74 | 11.07 |
| Microstate 4 | 8.99 | 4.51 | 3.46 | 24.00 |
| Microstate 5 | 10.00 | 6.73 | 3.34 | 38.71 |
| **Duration** |  |  |  |  |
| Microstate 1 | 2.21 | 1.03 | 0.53 | 5.38 |
| Microstate 2 | 2.20 | 0.85 | 0.73 | 4.24 |
| Microstate 3 | 2.39 | 1.09 | 0.72 | 5.04 |
| Microstate 4 | 2.77 | 1.42 | 0.65 | 7.21 |
| Microstate 5 | 2.57 | 1.09 | 1.01 | 6.90 |
| **Coverage** |  |  |  |  |
| Microstate 1 | 5.46 | 2.37 | 2.15 | 11.50 |
| Microstate 2 | 5.55 | 2.31 | 1.56 | 11.26 |
| Microstate 3 | 3.20 | 1.63 | 0.69 | 8.07 |
| Microstate 4 | 6.07 | 3.31 | 1.81 | 21.32 |
| Microstate 5 | 6.27 | 3.82 | 2.11 | 22.68 |
| **Occurrence** |  |  |  |  |
| Microstate 1 | 4.63 | 2.00 | 0.62 | 9.31 |
| Microstate 2 | 4.54 | 1.78 | 1.26 | 10.52 |
| Microstate 3 | 2.53 | 1.17 | 0.43 | 5.17 |
| Microstate 4 | 4.97 | 2.69 | 0.71 | 17.26 |
| Microstate 5 | 4.99 | 3.19 | 0.97 | 16.15 |

**Example** (corresponding to figure below)

We observed surprisingly high and sometimes near perfect correlations between the temporal property values of statistically significant comparisons. For example, microstate 5 coverage values showed a statistically significant comparison between four- and five-minute data durations. However, their values were correlated at *r* = .98. In contrast, microstate 5 coverage values were correlated at *r* = .70 for the non-significant one- versus five-minute comparison. Here, the higher correlation between the significant comparison suggests a more systematic change in values between data durations. In fact, computing the standard deviation of the paired difference scores across participants clarifies that relying on statistical significance may overlook nuanced, meaningful patterns in the data. Using the same example, the one- versus five-minute comparison showed a standard deviation of 2.14 but only 0.52 for the four- versus five-minute comparison, suggesting greater variability in the change scores of microstate 5 coverage for the non-significant relative to the significant comparison.

We also calculated the coefficient of variation between temporal property values derived from data of different durations for each participant. This allowed for a better understanding of individual variability in the stability of temporal properties. For example, the coefficient of variation ranged from 2.11 to 22.68 for microstate 5 coverage (mean = 6.27, standard deviation = 3.82), suggesting large variability in the stability of microstate 5 coverage between participants. That is, some participants showed small variations in microstate 5 coverage values derived from different data duration (e.g., 2.11) while other participants showed very large variations (e.g., 22.68). In future studies, the coefficient of variation can be used as method to quantify individual-level stability as a measure of interest that may relate to some aspect of behavior, or to exclude participants as having unstable or unreliable microstate metrics.


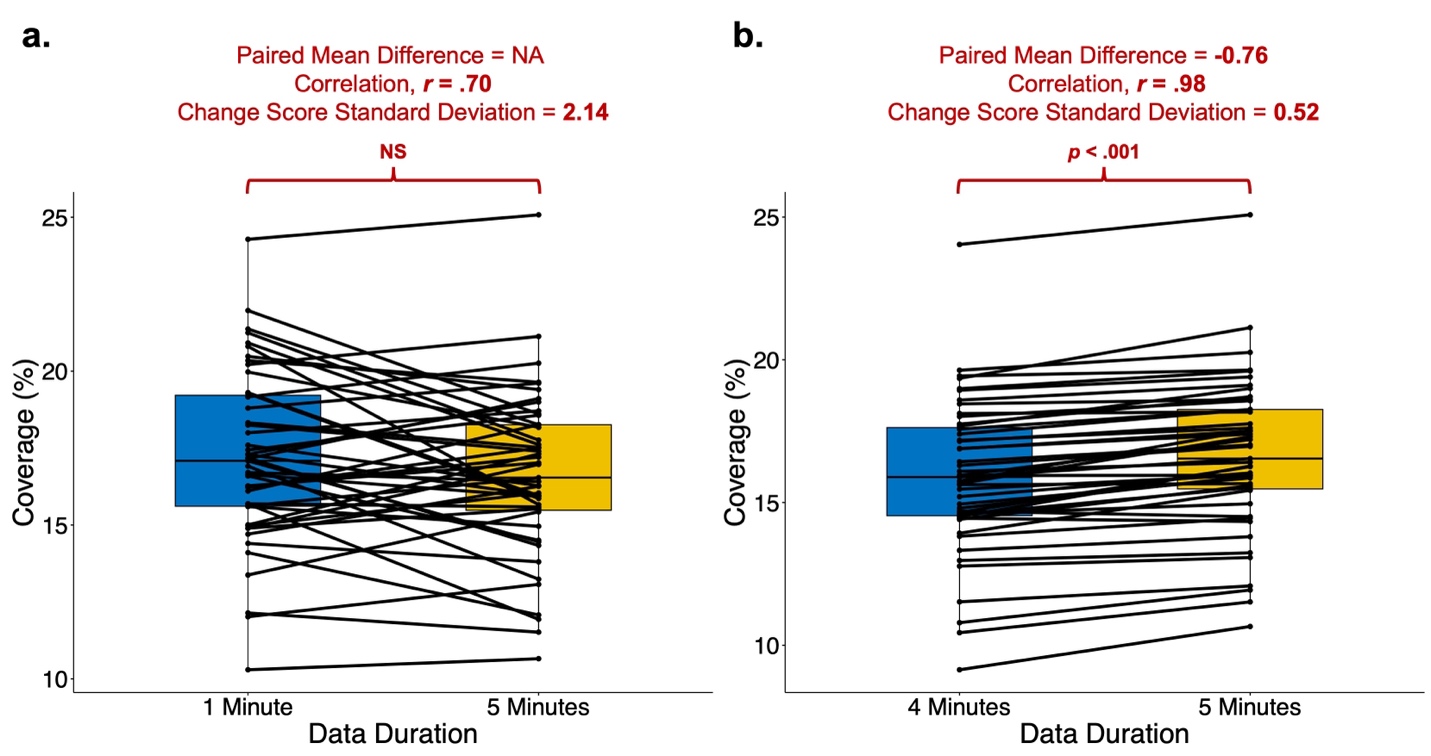


# Other

## S13. Software Versions

*E-Prime* Version 2.0.10.356

*NetStation Acquisition* Version 5.4.2 (r29917)

*MATLAB* Version R2022a

*EEGLAB* Version 2021.0

- *clean_rawdata* Version 2.7
- *ICLabel* Version 1.4
- *TBT* Version 2.6.1
- *firfilt* Version 2.6
- *MFFMatlabIO* Version 4.0

*Cartool* Version 4.11/7658

*AFNI* Version 22.1.10

*R* Version 4.2.2 (2022-10-31)

- *nlme* Version 3.1.164
- *effectsize* Version 0.8.6
- *dabestr* Version 0.3.0
- *rstatix* Version 0.7.2
- *emmeans* Version 1.8.9
- *MASS* Version 7.3-58.1
- *splithalfr* Version 2.2.0
- *ggplot2* Version 3.4.4
- ggpubr Version 0.6.0
